# Berberine and metabolic dysfunction-associated steatotic liver disease: a global analysis of molecular mechanisms, translational challenges and research frontiers

**DOI:** 10.3389/fnut.2026.1969017

**Published:** 2026-10-08

**Authors:** Fei Yu, Sihan Yin, Jiayu Zhu, Kewei Sun

**Affiliations:** 1 The First Clinical College of Chinese Medicine, Hunan University of Chinese Medicine, Changsha, Hunan, China; 2 Department of Hepatology, The First Hospital of Hunan University of Chinese Medicine, Changsha, Hunan, China

**Keywords:** berberine, bibliometric analysis, clinical efficacy, drug delivery, MASLD, molecular mechanism

## Abstract

**Objective:**

Metabolic dysfunction-associated steatotic liver disease (MASLD) has emerged as the most prevalent chronic liver disease worldwide. Berberine (BBR), a bioactive monomer derived from traditional Chinese medicine, demonstrates remarkable multi-target pharmacological properties in regulating glucose and lipid metabolism, ameliorating insulin resistance, attenuating inflammation and oxidative stress, and modulating the gut–liver axis. This study aims to systematically delineate the research landscape, thematic hotspots, and evolutionary trends of BBR in the treatment of MASLD through bibliometric analysis, and to provide an in-depth review of its underlying molecular mechanisms and clinical translational potential, thereby offering a valuable reference for future investigations.

**Methods:**

A dual-database literature search with cross-validation was conducted in the Web of Science Core Collection (WoSCC) and Scopus, utilizing refined retrieval strategies tailored to the research topic. Bibliometric analyses and network visualizations were performed using R (v4.5.1), VOSviewer (v1.6.19), and CiteSpace (v6.4.R1). Based on these scientometric findings, a comprehensive review of the identified mechanisms and clinical translational potential was subsequently undertaken.

**Results:**

A total of 341 and 624 records were retrieved from WoSCC and Scopus, respectively. Thematic hotspots converged on four major directions: (1) BBR regulates hepatic glucose and lipid metabolism via the AMP-activated protein kinase (AMPK)/Sirtuin 1 (SIRT1), Clock/Bmal1, and farnesoid X receptor (FXR)-Fibroblast Growth Factor 15 (FGF15) signaling pathways; (2) BBR attenuates MASLD progression through Nuclear factor erythroid 2-related factor 2 (Nrf2)/Antioxidant Response Element (ARE)-mediated antioxidant defense and suppression of nucleotide-binding oligomerization domain-like receptor family pyrin domain-containing 3 (NLRP3) inflammasome activation; (3) BBR reshapes gut microbiota architecture, restores mucosal barrier integrity, and modulates hepatic homeostasis via microbiota-derived metabolites along the gut–liver axis; and (4) the clinical efficacy of BBR in MASLD and the development of nano-delivery system-based formulations. Clinical evidence indicates that BBR ameliorates hepatic steatosis and improves metabolic profiles; however, its clinical translation remains constrained by poor oral bioavailability and substantial inter-patient variability in therapeutic response.

**Conclusion:**

BBR demonstrates a distinct therapeutic profile in MASLD management through the concerted modulation of hepatic metabolism, inflammation, oxidative stress, and the gut–liver axis. Future research should focus on developing innovative drug delivery systems to overcome bioavailability constraints, conducting large-scale, long-term randomized controlled trials to establish long-term efficacy and safety, and implementing precision medicine approaches guided by metabolic phenotyping.

## 1 Introduction

Metabolic dysfunction-associated steatotic liver disease (MASLD) is a metabolic stress induced liver injury closely associated with insulin resistance and genetic susceptibility. Its disease spectrum encompasses a progressive continuum ranging from simple hepatic steatosis to metabolic dysfunction-associated steatohepatitis (MASH), which may further advance to metabolic dysfunction-associated steatotic liver disease-fibrosis (MASLD-F), cirrhosis, and even hepatocellular carcinoma (HCC) (1). Beyond direct hepatic damage, MASLD accelerates the progression of coexisting liver diseases such as chronic hepatitis B (CHB) (2) and significantly increases the risk of cardiovascular disease, chronic kidney disease, and certain extrahepatic malignancies. Recent meta-analyses indicate that MASLD currently affects 38% of adults and 7–14% of children and adolescents worldwide (3), with adult prevalence projected to exceed 55.4% by 2040 (4). The escalating burden of MASLD is intimately linked to the global epidemic of obesity, type 2 diabetes mellitus, and metabolic syndrome. As the most prevalent chronic liver disease globally, MASLD now impacts approximately one-quarter of the world’s population, and its steadily rising prevalence poses a substantial threat to public health.

In 2024, the U.S. Food and Drug Administration (FDA) approved resmetirom (Rezdiffra), a selective thyroid hormone receptor-β (THR-β) agonist and the first targeted therapy for MASH, for adults with non-cirrhotic MASH at fibrosis stage F2 to F3 (5). Phase III MAESTRO-MASH trial confirmed the histological improvements induced by resmetirom in patients with MASH accompanied by liver fibrosis (6, 7). Subsequently, in 2025, the FDA issued accelerated conditional approval for once-weekly subcutaneous semaglutide 2.4 mg, a glucagon-like peptide-1 receptor agonist (GLP-1 RA), for the treatment of adults with MASH and moderate-to-advanced liver fibrosis (F2–F3) (8). Although these two drug classes represent historic milestones, their clinical use remains limited by practical constraints, including inadequate response rates and uncertain long-term safety profiles.

Berberine (BBR), a quaternary ammonium salt is quinoline alkaloid with the chemical formula of C20H18NO4+, is a natural composite derived from several plants, especially *Berberis vulgaris* L, which is from the Berberidaceae family (9). It has been demonstrated that the hepatic tissues are the major target of BBR since the liver has the highest con-centration of BBR metabolites (approximately 70 times as large as its concentration in serum) (10). Modern pharmacological investigations have revealed that BBR exerts multifaceted bioactivities, including amelioration of insulin resistance, regulation of glucose and lipid metabolism, attenuation of oxidative stress and inflammation, and modulation of the gut microbiota, thereby demonstrating broad therapeutic potential across metabolic disorders. A systematic elucidation of the therapeutic efficacy and underlying molecular mechanisms of BBR against MASLD will provide robust theoretical underpinnings and translational insights for basic research, clinical practice, and novel drug development.

Although research on BBR for MASLD has expanded exponentially in recent years, existing reviews have predominantly been traditional narrative overviews lacking quantitative, visualized analyses of the field’s evolutionary trajectory, thematic clustering, and emerging frontiers. Accordingly, the present study employs a bibliometric approach to systematically retrieve and analyze relevant literature from the WoSCC, Scopus, and PubMed databases, aiming to comprehensively map the research landscape, identify thematic hotspots, synthesize key clinical evidence, and forecast developmental trends in BBR-mediated MASLD treatment. Through this analysis, we seek to pinpoint research priorities and furnish a scientific reference for subsequent basic investigations and clinical translation.

## 2 Materials and methods

### 2.1 Data collection

The WoSCC and Scopus databases were selected as the primary sources for the comprehensive bibliometric analysis, with data retrieved on April 1, 2026. After removing irrelevant records, 166 eligible publications were identified from WoSCC and 334 from Scopus. Additionally, relevant clinical trial data were retrieved from PubMed. Detailed search strategies are provided in Annex 1. This study adopted an approach of independent analysis with mutual corroboration: WoSCC served as the primary data source for bibliometric analysis; Scopus was employed to explore research trends, hotspots, and thematic evolution, aiming to supplement and cross-validate the WoSCC findings; and PubMed was dedicated to the retrieval and evaluation of clinical trials, with a specific focus on clinical translational evidence of BBR in MASLD treatment.

### 2.2 Data analysis

This study utilized OriginPro 2025 software to analyze annual publication trends and employed R software (version 4.5.1) with the bibliometrix package (version 5.0.1), VOSviewer (version 1.6.19), and CiteSpace (version 6.4.R1) for bibliometric data analysis and visualization. Journal impact factors were retrieved from the 2025 Journal Citation Reports (JCR). Each database was processed separately. VOSviewer was used to generate network maps of country- and institution-level co-authorship, document co-citation, and keyword co-occurrence. In co-authorship analyses, only countries with ≥ one publications and institutions with ≥2 publications were retained. For co-citation analysis, documents cited ≥13 times were included. To standardize the keyword pool, terms appearing in ≥2 WoSCC records were admitted; generic phrases such as “Berberine” and “MASLD” (and their synonyms) were excluded. CiteSpace’s time-slicing function was employed to identify emergent research fronts. By integrating these approaches, we have delineated a research framework for berberine in the treatment of MASLD.

## 3 Results

### 3.1 General analysis

A total of 166 publications were retrieved from WoSCC. As shown in Figure 1, the annual publication output demonstrated an overall upward trend from 2012 to 2026. Furthermore, Figure 2A illustrates that China maintained the most extensive international collaboration in this field. The collaboration network highlighted Fudan University (*n* = 9) and Guangzhou University of Chinese Medicine (*n* = 7) as leading institutions with notable research productivity and collaborative activities (Figure 2B); detailed data are provided in Annex 2. Seven clinical research articles were retrieved from PubMed. Additionally, from among the 166 WoSCC records, 8 clinical studies were ultimately included following the selection procedure outlined in Figure 3, after excluding duplicates.

**Figure 1:**
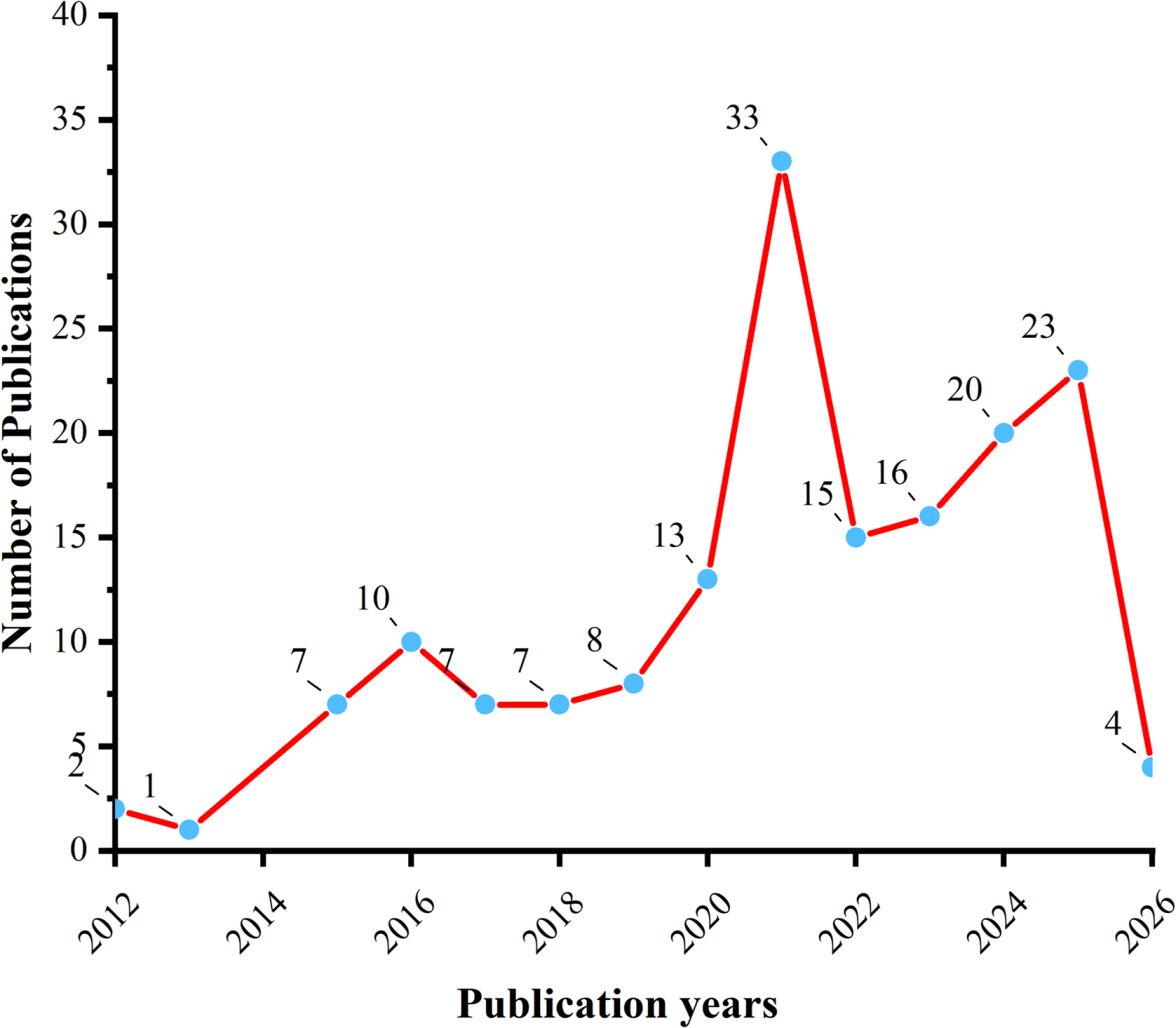
Annual publication output trends in Berberine for MASLD.

**Figure 2:**
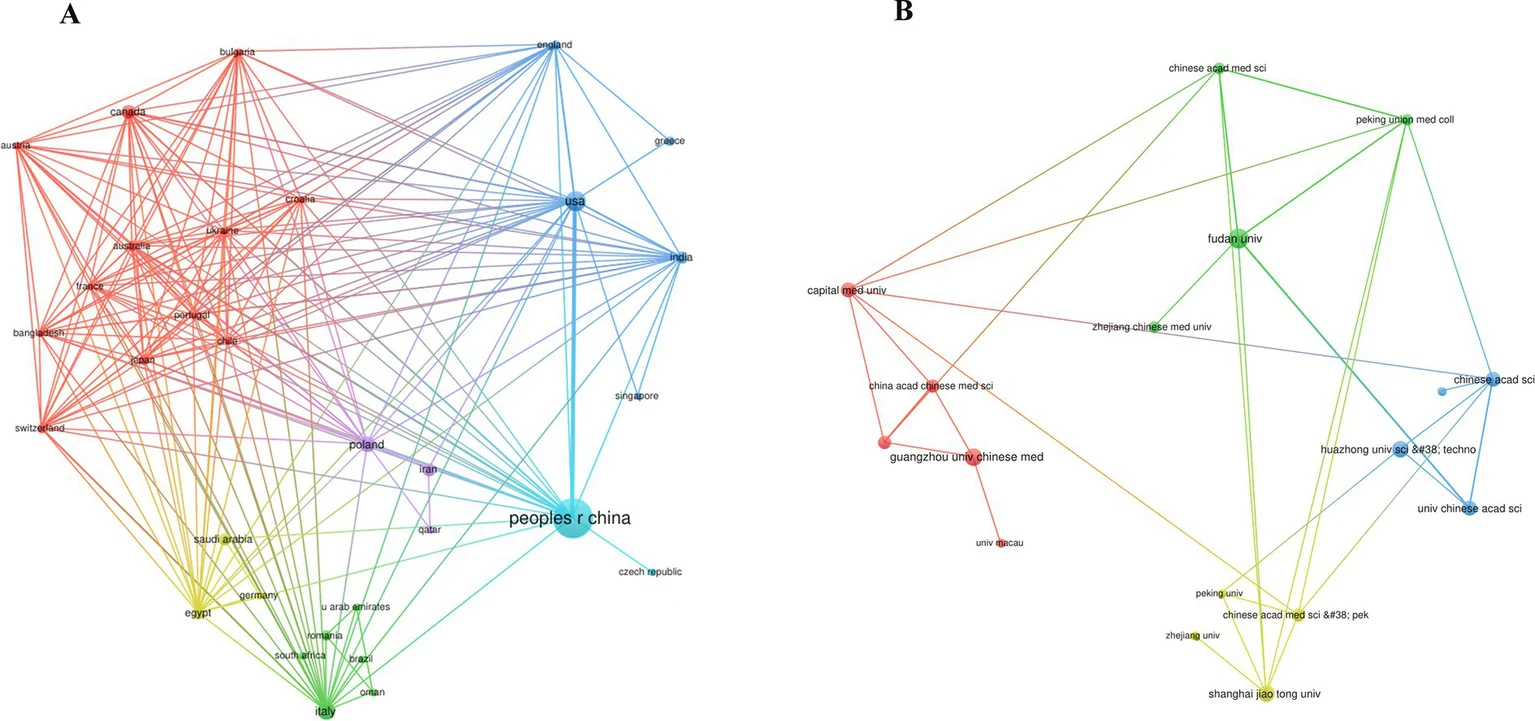
Countries/regions and institutional collaboration maps of Berberine for MASLD. **(A)** International collaboration map. **(B)** Institutional collaboration map.

**Figure 3:**
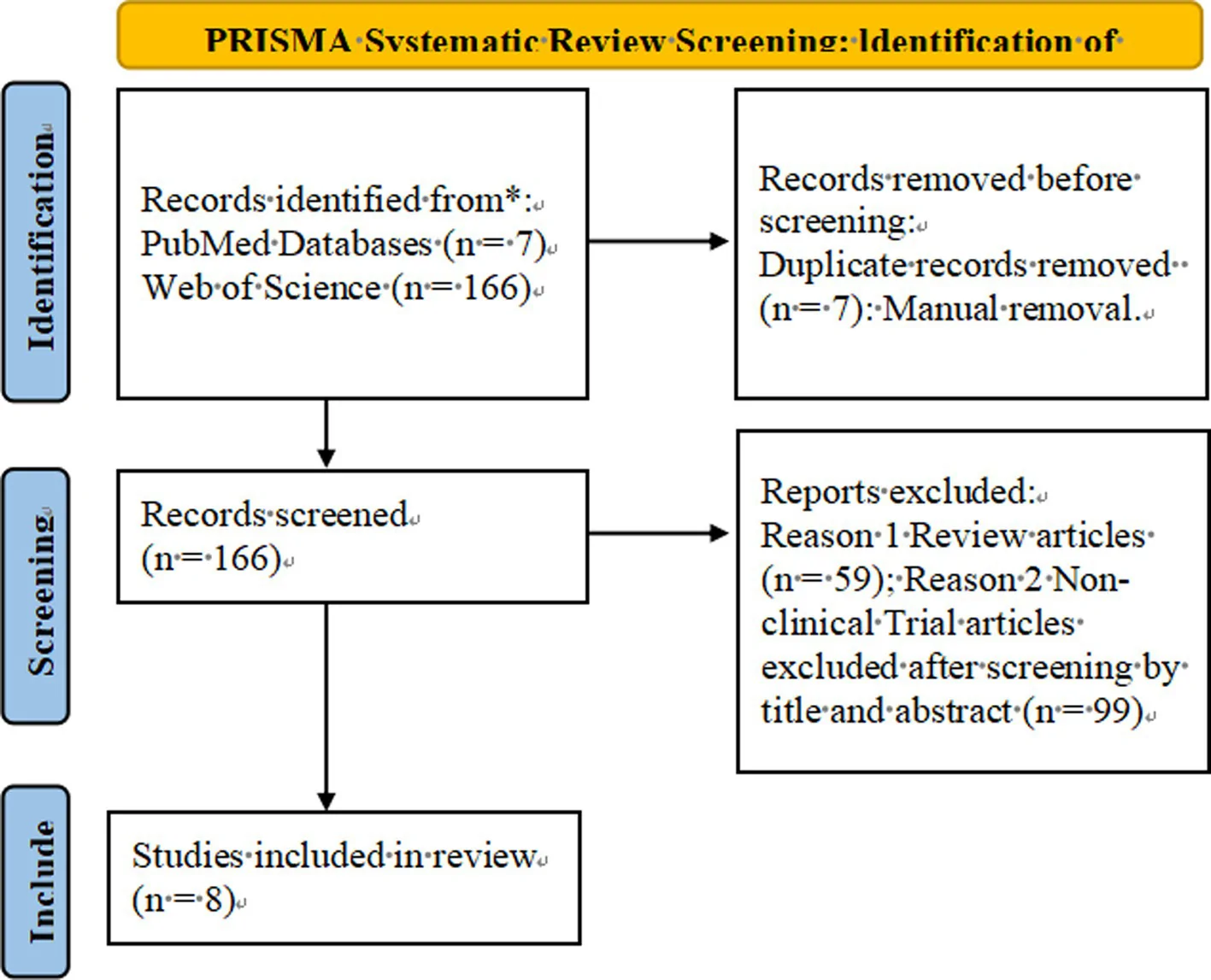
PRISMA flow diagram for the study selection process.

### 3.2 Journal analysis and visualization

The bibliometrix and ggplot2 packages in R software (version 4.4.1) were utilized to analyze the journals publishing research on BBR in MASLD. Additionally, VOSviewer (version 1.6.19) was employed for co-citation journal analysis. The results indicated that the 166 WoSCC-indexed articles were distributed across 96 distinct academic journals (Annex 3).

Regarding journal publication patterns, the two most prolific journals were Frontiers in Pharmacology (*n* = 12, IF = 4.8) and Nutrients (*n* = 10, IF = 5.0), as shown in Table 1 and Figure 4A.

**Table 1:** Top 10 journals with the most published articles.

Journal	Documents	IF (2025)	Cites
Frontiers in Pharmacology	12	4.8	230
Nutrients	10	5	265
Journal of Ethnopharmacology	7	5.4	133
International Journal of Molecular Sciences	6	4.9	208
Biomedicine and Pharmacotherapy	4	7.5	200
Experimental and Therapeutic Medicine	4	2.3	64
PLoS One	4	2.6	301
Chinese Medicine	3	5.7	10
Journal of Translational Medicine	3	7.5	77
Molecules	3	4.6	120

**Figure 4:**
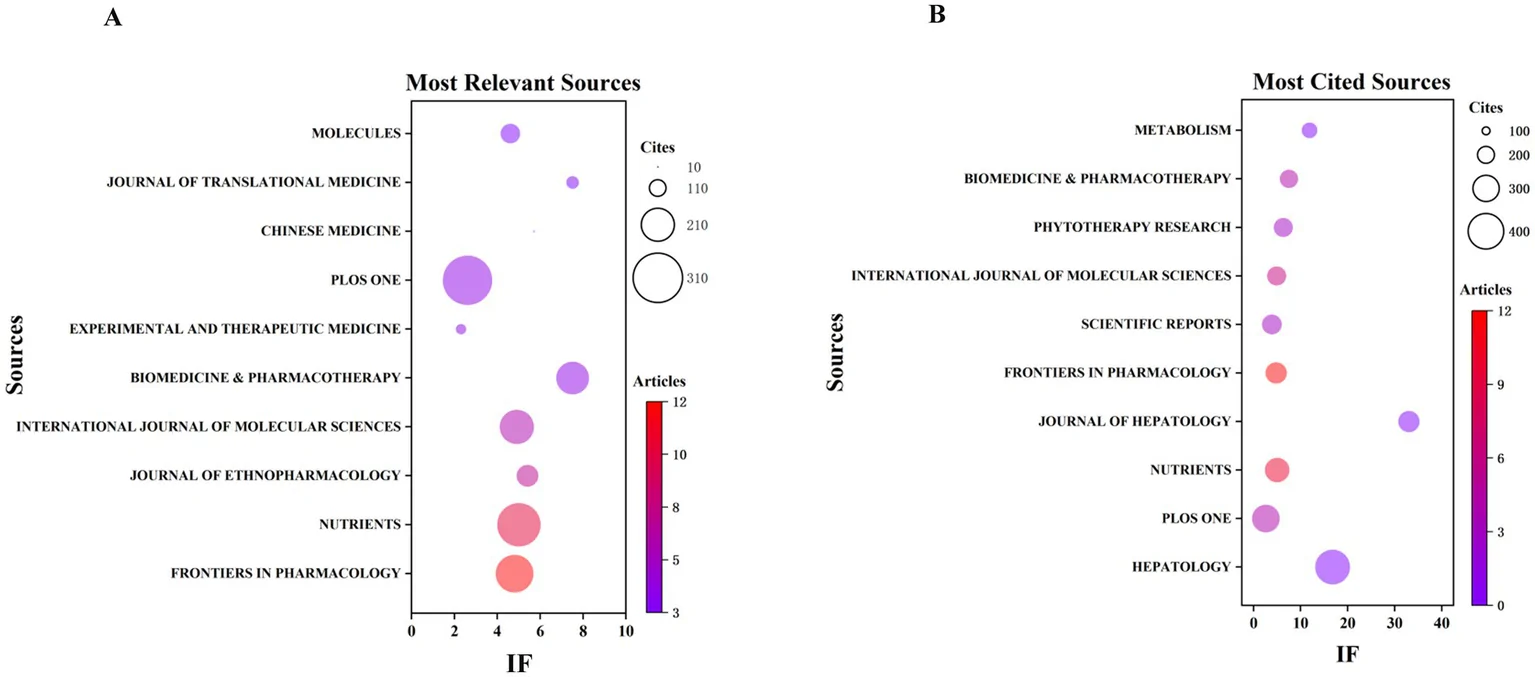
Journal with the largest number of articles published and the journal with the largest number of citations. **(A)** Journal with the largest number of articles published. **(B)** Journals with the largest number of citations.

Beyond publication volume, we also examined journals with high citation impact. As presented in Table 2 and Figure 4B, Hepatology ranked first in citation frequency (*n* = 377, IF = 16.8), followed by PLOS ONE (*n* = 301, IF = 2.6), Nutrients (*n* = 265, IF = 5.0), and the Journal of Hepatology (*n* = 232, IF = 33.0). Integrating both publication output and citation metrics, Nutrients emerged as a prominent journal for both publishing and citing articles in this field.

**Table 2:** Top 10 journals with the most cited journals.

Journal	Cites	IF (2025)	Documents
Hepatology	377	16.8	0
PLoS One	301	2.6	4
Nutrients	265	5	10
Journal of Hepatology	232	33	0
Frontiers in Pharmacology	230	4.8	12
Scientific Reports	215	3.9	3
International Journal of Molecular Sciences	208	4.9	6
Phytotherapy Research	207	6.3	3
Biomedicine and Pharmacotherapy	200	7.5	4
Metabolism	169	11.9	0

Co-citation journal visualization analysis (Figure 5) further demonstrated that Hepatology, PLOS ONE, and Nutrients constitute highly influential journals in the field of BBR for MASLD. Collectively, these data indicate that this research domain has generated substantial output in high-impact journals, reflecting broad international recognition and underscoring the significant academic value and practical relevance of this research direction.

**Figure 5:**
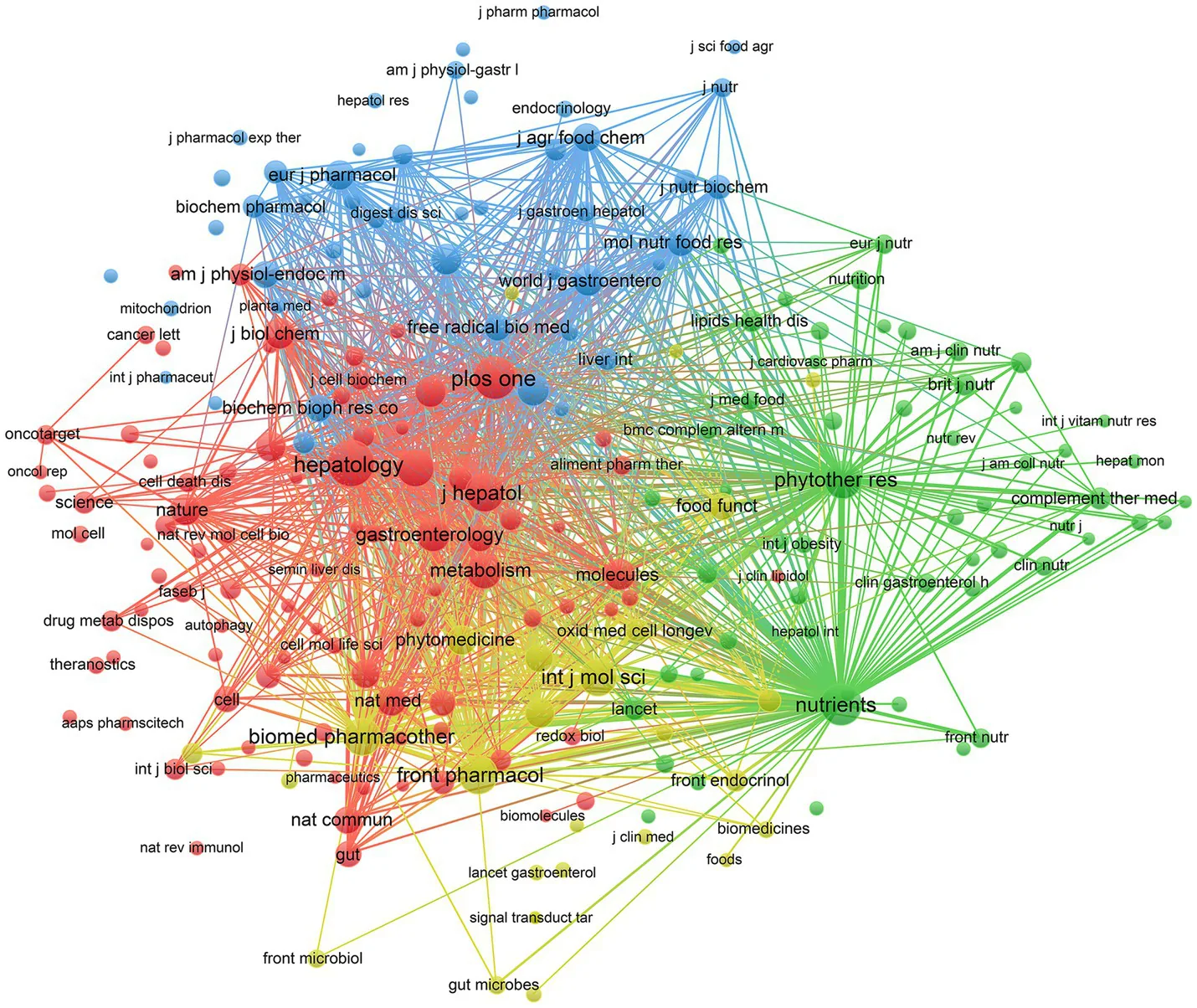
Co-cited journals involved in Berberine for MASLD.

### 3.3 Most cited references and reference burst

Using the bibliometrix package in R, we identified the 20 most-cited articles in the field of BBR for MASLD. In WoSCC, each of these articles received more than 82 citations, spanning 15 distinct journals; in Scopus, each accumulated over 198 citations, distributed across 18 different journals (Table 3). Through comprehensive analysis, these 20 papers were categorized into four thematic clusters: (1) molecular mechanisms of BBR in regulating hepatic glucose and lipid metabolism; (2) the role and mechanisms of BBR in alleviating MASLD-related inflammation and pathological progression; (3) gut–liver axis–mediated mechanisms of BBR in MASLD; and (4) clinical investigations and formulation development of BBR for MASLD.

**Table 3:** Top 20 cited references related to berberine for MASLD.

WoSCC database				Scopus database			
Paper	DOI	Total Citations	TC per Year	Paper	DOI	Total Citations	TC per Year
Song DY, 2020, Front Med-PRC	10.1007/s11684-019-0724-6	406	58.00	Leung C, 2016, Nat Rev Gastroenterol Hepatol	10.1038/nrgastro.2016.85	926	84.18
Zhu XP, 2019, Free Radical Bio Med	10.1016/j.freeradbiomed.2019.06.019	228	28.50	Wu J, 2021, Protein Cell	10.1007/s13238-020-00814-7	464	77.33
Yan HM, 2015, PLoS One	10.1371/journal.pone.0134172	196	16.33	Song D, 2020, Front Med	10.1007/s11684-019-0724-6	460	65.71
Mishra S, 2019, Cell Mol Life Sci	10.1007/s00018-019-03053-0	195	24.38	Feng X, 2019, Theranostics	10.7150/thno.30787	401	50.13
Xu XM, 2021, Biomed Pharmacother	10.1016/j.biopha.2020.110984	188	31.33	Feng W, 2019, Pharmacol Res	10.1016/j.phrs.2019.02.024	344	43.00
Yan TT, 2020, Acta Pharm Sin B	10.1016/j.apsb.2019.11.017	187	26.71	Spahis S, 2017, Antioxid Redox Signal	10.1089/ars.2016.6776	344	34.40
Sun RB, 2017, MOL PHARMACOL	10.1124/mol.116.106617	160	16.00	Imenshahidi M, 2016, Phytother Res	10.1002/ptr.5693	335	30.45
Man SL, 2021, Eur J Med Chem	10.1016/j.ejmech.2021.113690	141	23.50	Li S, 2016, Oxidative Med Cell Longevity	10.1155/2016/4234061	332	30.18
Cicero AFG, 2018, Nutrients	10.3390/nu10091153	135	15.00	Imenshahidi M, 2019, Phytother Res	10.1002/ptr.6252	330	41.25
Shabalala SC, 2020, Biomed Pharmacother	10.1016/j.biopha.2020.110785	121	17.29	Nassir F, 2014, Int J Mol Sci	10.3390/ijms15058713	319	24.54
Rafiei H, 2019, Nutrients	10.3390/nu11030541	116	14.50	Srivastava RAK, 2012, J Lipid Res	10.1194/jlr. R025882	277	18.47
Yuan XL, 2015, J Transl Med	10.1186/s12967-015-0383-6	106	8.83	Popov Y, 2009, Hepatology	10.1002/hep.23123	276	15.33
Mai WJ, 2020, Front Pharmacol	10.3389/fphar.2020.00185	99	14.14	Iside C, 2020, Front Pharmacol	10.3389/fphar.2020.01225	259	37.00
Zhong MY, 2022, Food Funct	10.1039/d2fo01009g	95	19.00	Zhu X, 2019, Free Radic Biol Med	10.1016/j.freeradbiomed.2019.06.019	244	30.50
Harrison SA, 2021, Nat Commun	10.1038/s41467-021-25701-5	95	15.83	Cicero AFG, 2017, Arch Med Sci	10.5114/aoms.2017.69326	227	22.70
Guo T, 2016, Sci REP-UK	10.1038/srep22612	94	8.55	Yan H-M, 2015, PLoS One	10.1371/journal.pone.0134172	220	18.33
Qiang XY, 2016, Biochem Bioph Res Co	10.1016/j.bbrc.2016.03.019	90	8.18	Xu X, 2021, Biomed Pharmacother	10.1016/j.biopha.2020.110984	218	36.33
Zhang ZG, 2016, Sci REP-UK	10.1038/srep20848	86	7.82	Mishra S, 2019, Cell Mol Life Sci	10.1007/s00018-019-03053-0	210	26.25
Wang Y, 2020, PLoS One	10.1371/journal.pone.0232630	83	11.86	Bagherniya M, 2018, Pharmacol Res	10.1016/j.phrs.2017.12.020	205	22.78
Lu JZ, 2021, Front Pharmacol	10.3389/fphar.2021.668418	82	13.67	Yan T, 2020, Acta Pharm Sin B	10.1016/j.apsb.2019.11.017	198	28.29

To identify prominent citation burst patterns in the field of BBR for MASLD, CiteSpace was applied to the WoSCC database, detecting 18 references with significant citation bursts based on predefined criteria (Figure 6). The titles and corresponding DOIs of these references are listed in Annex 4. The burst intensities of these 18 documents ranged from 2.9 to 9.26. The top three were: Efficacy of Berberine in Patients with Non-Alcoholic Fatty Liver Disease (strength: 9.26); Berberine in Non-Alcoholic Fatty Liver Disease-A Review (strength: 4.35); and Berberine ameliorates hepatic steatosis and suppresses liver and adipose tissue inflammation in mice with diet-induced obesity (strength: 3.89).

**Figure 6:**
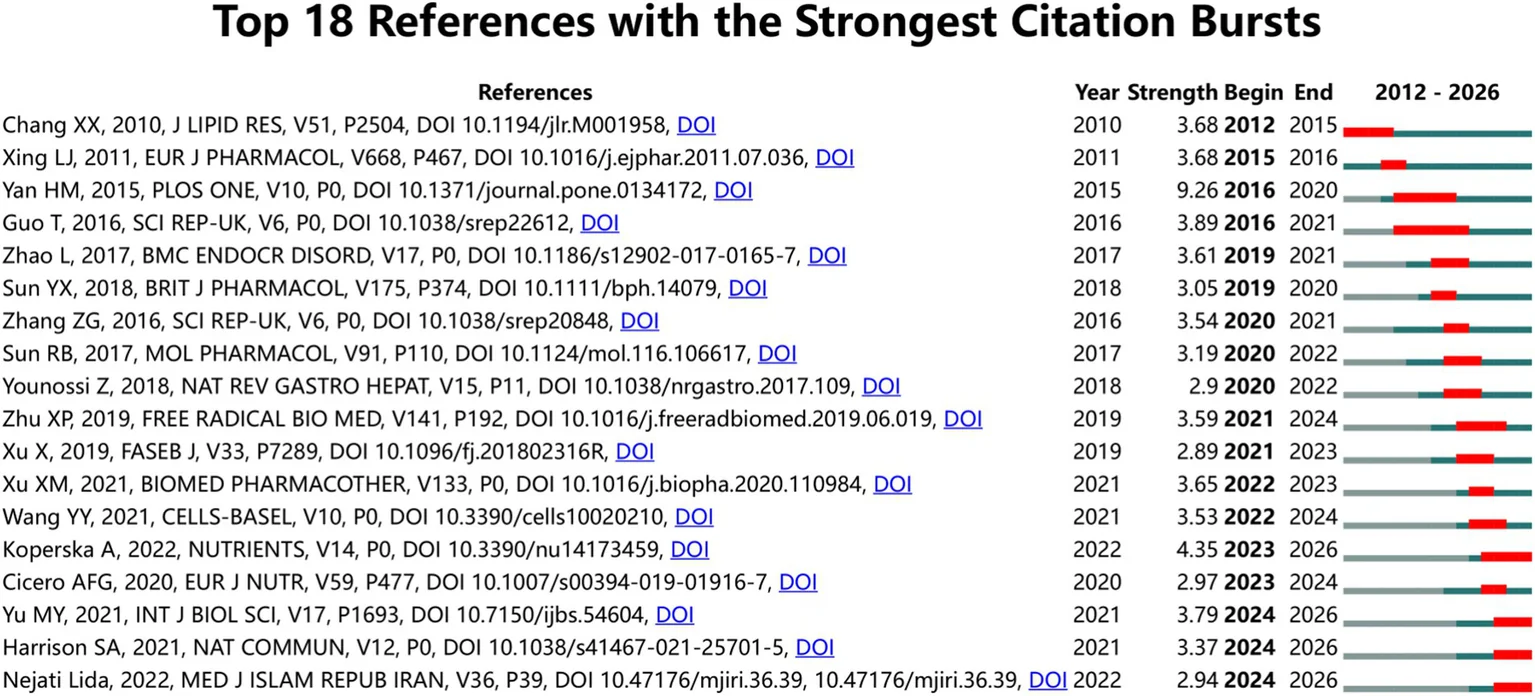
Top 18 references with the strongest citation bursts in Berberine for MASLD.

These findings reveal that core research in this field centers on molecular pathways of glucose and lipid metabolism and multi-target intervention against MASLD pathological progression. Notably, gut–liver axis modulation represents an emerging direction, whereas clinical efficacy validation and formulation development constitute critical translational research priorities.

### 3.4 Keyword clusters and evolution of themes

Keyword clustering offers a rapid methodological approach to identifying principal themes and research directions within a given field. In this study VOSviewer was employed to extract 920 keywords from WoSCC and 6,248 keywords from Scopus. Table 4 Presents the top 20 keywords. In WoSCC the most frequently occurring keyword was “insulin resistance” (*n* = 50) followed by “oxidative stress” (*n* = 34) and “gut microbiota” (*n* = 30). In Scopus the most frequent keyword was “human” (*n* = 250) followed by “nonhuman” (*n* = 239) and “review” (*n* = 172).

**Table 4:** The top 20 keywords.

WoSCC database			Scopus database		
Rank	Keywords	Count	Rank	Keywords	Count
1	insulin resistance	50	1	human	250
2	oxidative stress	34	2	nonhuman	239
3	gut microbiota	30	3	review	172
4	activated protein-kinase	26	4	unclassified drug	140
5	obesity	26	5	metabolism	139
6	hepatic steatosis	25	6	animal	130
7	inflammation	25	7	article	123
8	mechanism	23	8	controlled study	110
9	expression	22	9	drug effect	108
10	lipid metabolism	22	10	lipid metabolism	108
11	activation	18	11	insulin resistance	107
12	mice	16	12	male	101
13	diet	14	13	obesity	94
14	acid	13	14	inflammation	89
15	fibrosis	13	15	animal experiment	87
16	metabolism	13	16	oxidative stress	86
17	natural products	13	17	curcumin	83
18	steatosis	13	18	animal model	82
19	amp kinase	12	19	non insulin dependent diabetes mellitus	82
20	traditional Chinese medicine	11	20	triacylglycerol	79

Based on these data, we selected 200 keywords with a minimum occurrence of 2 from WoSCC and 207 keywords with a minimum occurrence of 16 from Scopus to generate keyword cluster maps (Figures 7A,B). In WoSCC, five major clusters were identified and analyzed, each comprising no fewer than 26 keywords: Cluster 1 (red): Gut Microbiota and Inflammatory–Immune Regulation—this cluster comprised 68 keywords, including “gut microbiota,” “inflammation,” and “expression”; Cluster 2 (green): Mechanisms of Berberine in Lipid Metabolism Regulation—encompassing 39 keywords such as “mechanism,” “natural products,” and “steatosis”; Cluster 3 (blue): Synergistic Amelioration of Insulin Resistance and Oxidative Stress—comprising 34 keywords including “insulin resistance,” “oxidative stress,” and “signaling pathway”; Cluster 4 (yellow): Hepatic Energy Metabolism Modulation by Berberine via Autophagy–Apoptosis Balance—containing 33 keywords such as “activated protein-kinase,” “obesity,” and “diet-induced obesity”; and Cluster 5 (purple): Integrated Management of Hepatic Steatosis and Metabolic Syndrome—encompassing 26 keywords including “hepatic steatosis,” “lipid metabolism,” and “metabolic syndrome.” In Scopus, three major clusters were identified and analyzed, each comprising no fewer than 4 keywords: Cluster 1 (red): Core Pathological Mechanisms of Berberine in MASLD Regulation—Intervention in the Metabolic Dysregulation–Oxidative Stress–Inflammation Axis; this cluster contained 102 keywords, including “human,” “nonhuman,” and “review.” Cluster 2 (green): Technical Methodologies and Multi-Target Mechanistic Analysis of Berberine in MASLD Treatment—including 82 keywords such as “metabolism,” “animal,” and “article.” Cluster 3 (blue): Clinical Translation and Population-Associated Studies of Berberine for MASLD—comprising 4 keywords, including “drug therapy” and “adult” (complete list provided in Annex 5).

**Figure 7:**
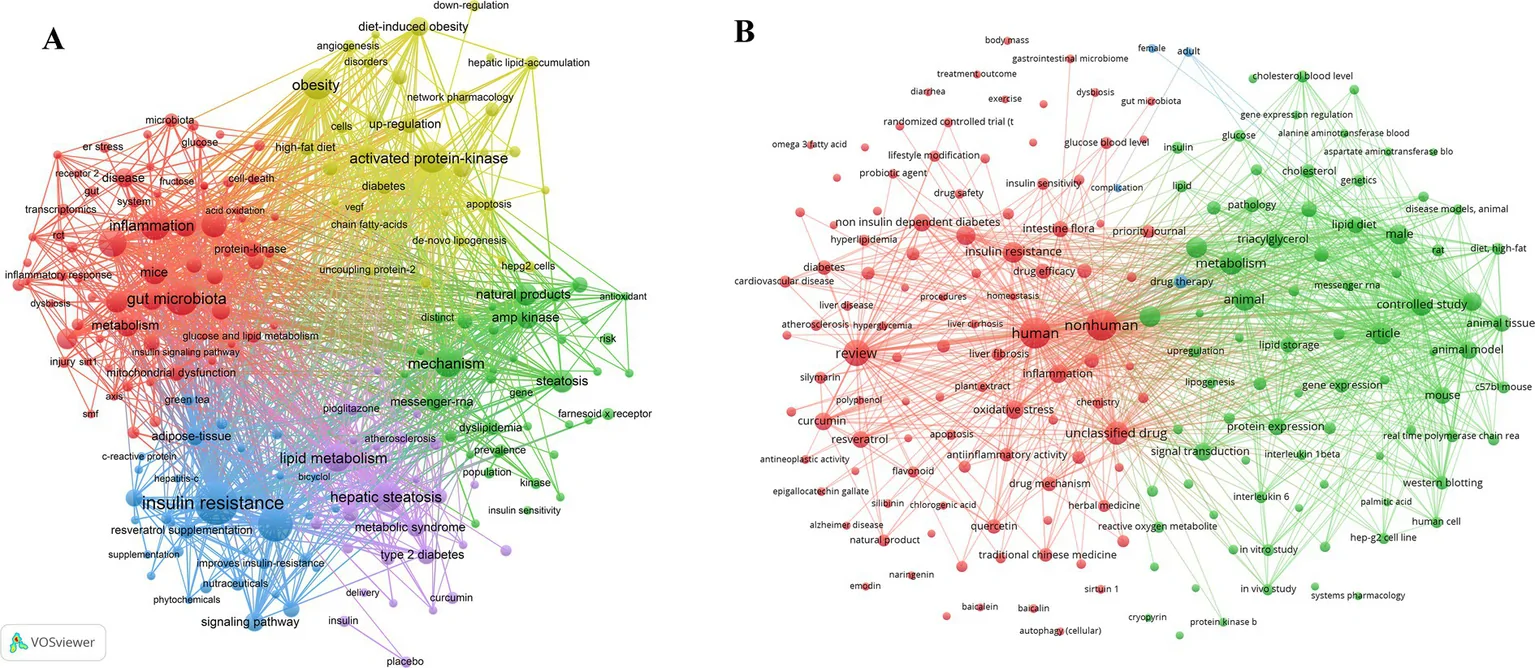
Keyword co-occurrence map of publications on Berberine for MASLD [**(A)** from the WoSCC; **(B)** from the Scopus database].

To analyze developmental trends in both databases from 2011 to the first quarter of 2026, we constructed dynamic thematic evolution maps using the bibliometrix package within the R programming environment (Figures 8A,B). Owing to limited publication output and scattered keyword distribution during 2011–2015—which precluded the establishment of a discernible research framework—the effective starting year for trend analysis was set at 2016. The research trajectory evolved sequentially through three phases: (i) fundamental mechanistic exploration (2016–2019), focusing on drug comparisons and pathophysiological investigations; (ii) molecular mechanism deepening (2020–2023), centering on the core effects of berberine with in-depth exploration of molecular targets and signaling pathways related to oxidative stress and insulin resistance; and (iii) clinical translation expansion (2024–2026), shifting toward double-blind clinical trials, combination therapeutic strategies, and disease nomenclature standardization. Looking ahead, the thematic evolution maps indicate that future research on berberine for MASLD will be closely linked to the construction of high-quality clinical evidence represented by multicenter randomized controlled trials; individualized precision therapy exemplified by patient-stratified dose optimization; innovative combination strategies involving synergistic administration with natural products or targeted agents; pharmaceutical technology advancements represented by novel drug delivery systems aimed at improving bioavailability; and differentiated interventions tailored to distinct pathological stages of MASLD. Collectively, these developments will propel the field from mechanistic research toward in-depth clinical application.

**Figure 8:**
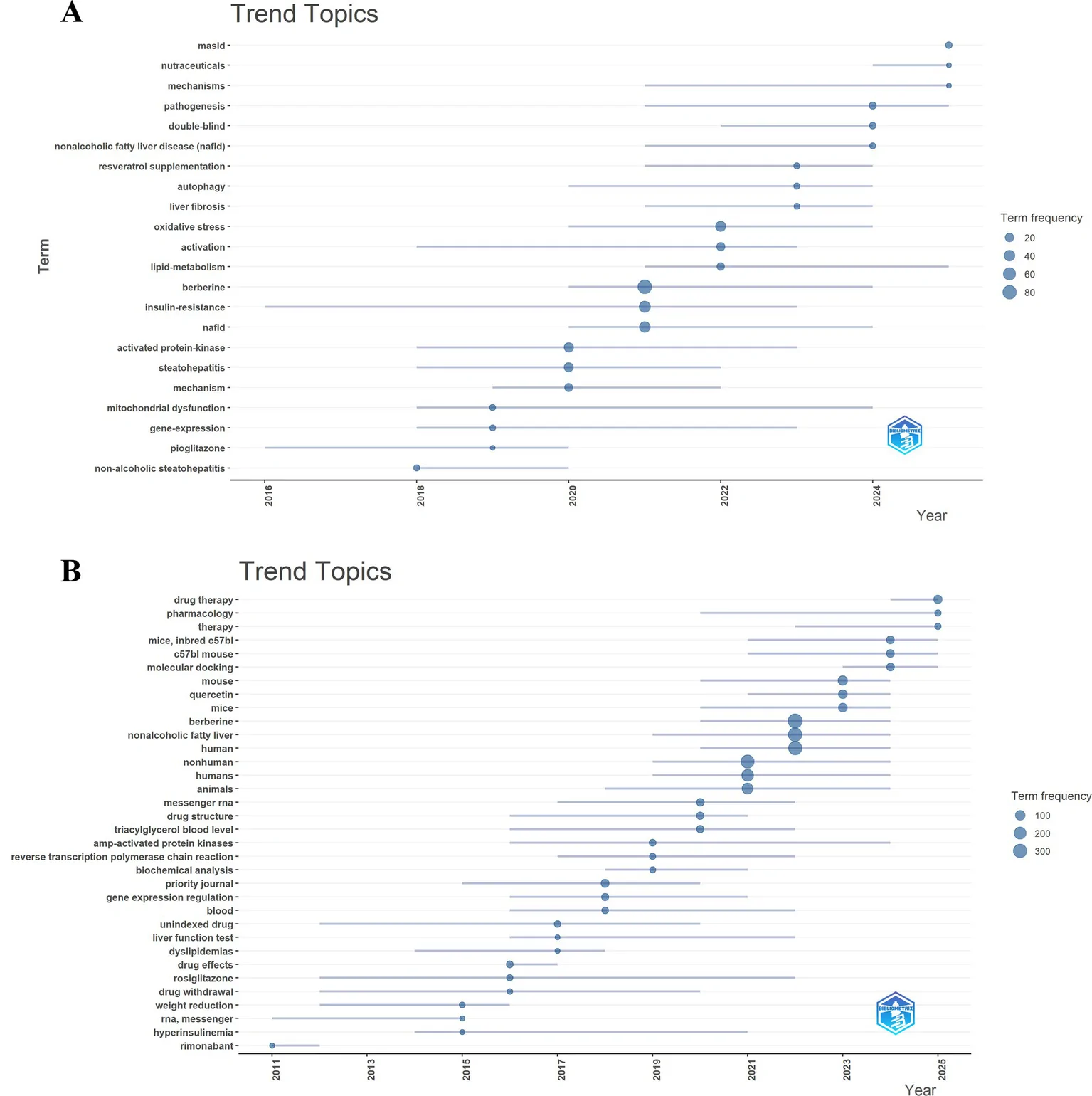
Trend topics on Berberine for MASLD [**(A)** from the WoSCC; **(B)** from the Scopus database].

### 3.5 Comprehensive analysis of hotspots

In summary, the aforementioned analyses reveal that emerging research hotspots in berberine for MASLD treatment concentrate in four principal areas: (1) molecular mechanisms and signaling pathways underlying berberine-mediated regulation of hepatic glucose and lipid metabolism; (2) the effects and mechanisms of berberine in attenuating the pathological progression of MASLD from simple steatosis to MASH and hepatic fibrosis; (3) gut–liver axis–mediated mechanisms by which berberine modulates MASLD; and (4) clinical investigations and formulation development of berberine for MASLD. To facilitate the translation of berberine from experimental research to clinical application in MASLD, future studies should prioritize multicenter, large-sample, randomized controlled trials with long-term follow-up; optimize dosing regimens and develop novel formulations to enhance bioavailability; identify predictive biomarkers of therapeutic efficacy; and explore combination therapeutic strategies targeting the gut–liver axis and metabolic–inflammatory networks. Such efforts will be instrumental in advancing berberine as an effective agent for the precision treatment of MASLD.

In contrast to existing reviews, the present manuscript offers three unique contributions. First, it adopts an innovative perspective by integrating bibliometric big data analysis, pharmacological mechanism synthesis, and formulation translational assessment to construct a comprehensive research panorama. Second, it delivers innovative content through a systematic summary of berberine nanoformulations, bioavailability optimization strategies, and translational bottlenecks, areas that have been insufficiently explored in prior reviews. Third, it provides innovative conclusions whereby quantitative analysis objectively identifies research gaps and emerging frontiers, offering insights unattainable through conventional narrative approaches.

Beyond the above mentioned advances, the present work also pinpoints distinctive knowledge gaps, mechanistic considerations, and translational implications uncovered through this systematic literature synthesis. In terms of knowledge gaps, we highlight the scarcity of high quality multicenter long term clinical evidence, unresolved inter-individual heterogeneity in therapeutic responses to berberine, and multiple unaddressed bottlenecks restricting the clinical translation of berberine nano-formulations. For mechanistic insights, we underscore that most mechanistic observations are derived from cellular and animal experiments, whereas human validated molecular targets and causal evidence for the gut-liver-axis effects remain limited. From a translational perspective, our analysis emphasizes urgent priorities including optimized clinical trial design, formulation oriented technical breakthroughs, and stratified population oriented intervention strategies, which can inform the design of subsequent basic experiments and clinical investigations on berberine for MASLD.

## 4 Discussion

### 4.1 General information

A total of 166 relevant articles published between 2012 and the first quarter of 2026 were collected from the WoSCC, whereas 334 articles published between 2011 and the first quarter of 2026 were retrieved from Scopus. Collectively, publication output in this field exhibited a sustained upward trend overall. Notably, a marked decline was observed during 2021–2022. This downturn may be partially attributable to disruptions in clinical trials and laboratory operations caused by the Coronavirus Disease 2019 (COVID-19) pandemic, which delayed numerous research projects and temporarily suppressed publication output in 2022 before a gradual recovery ensued. Based on publication volume, Frontiers in Pharmacology and Nutrients emerged as the two most prolific journals. Regarding citation impact, Hepatology held the preeminent position by a substantial margin, reaffirming its standing as the principal vehicle for disseminating influential research in this domain.

### 4.2 Hotspots and development trends

The pathogenetic understanding of MASLD has evolved from the classical “two-hit” hypothesis to a contemporary “multiple-hit” framework. The pathogenesis underlying MASLD initiation and progression is elaborated comprehensively in Figure 9. Beyond these mechanistic insights, the therapeutic paradigm for MASLD has shifted from an extended era of symptomatic management reliant solely on lifestyle interventions to the current era of precision pharmacotherapy targeting core pathological drivers. Traditional Chinese medicine (TCM) has accumulated extensive theoretical knowledge and practical experience in MASLD prevention and management, offering distinctive therapeutic advantages. Recently, bioactive monomers derived from TCM have garnered substantial research attention owing to their well-defined chemical identities, clearly elucidated mechanisms of action, and controllable pharmacological profiles. Among these, berberine has emerged as one of the most promising natural product candidates for MASLD treatment, distinguished by its multifaceted pharmacological activities encompassing the regulation of glucose and lipid metabolism, amelioration of insulin resistance, suppression of oxidative stress and inflammation, and modulation of the gut microbiota and gut–liver axis.

**Figure 9:**
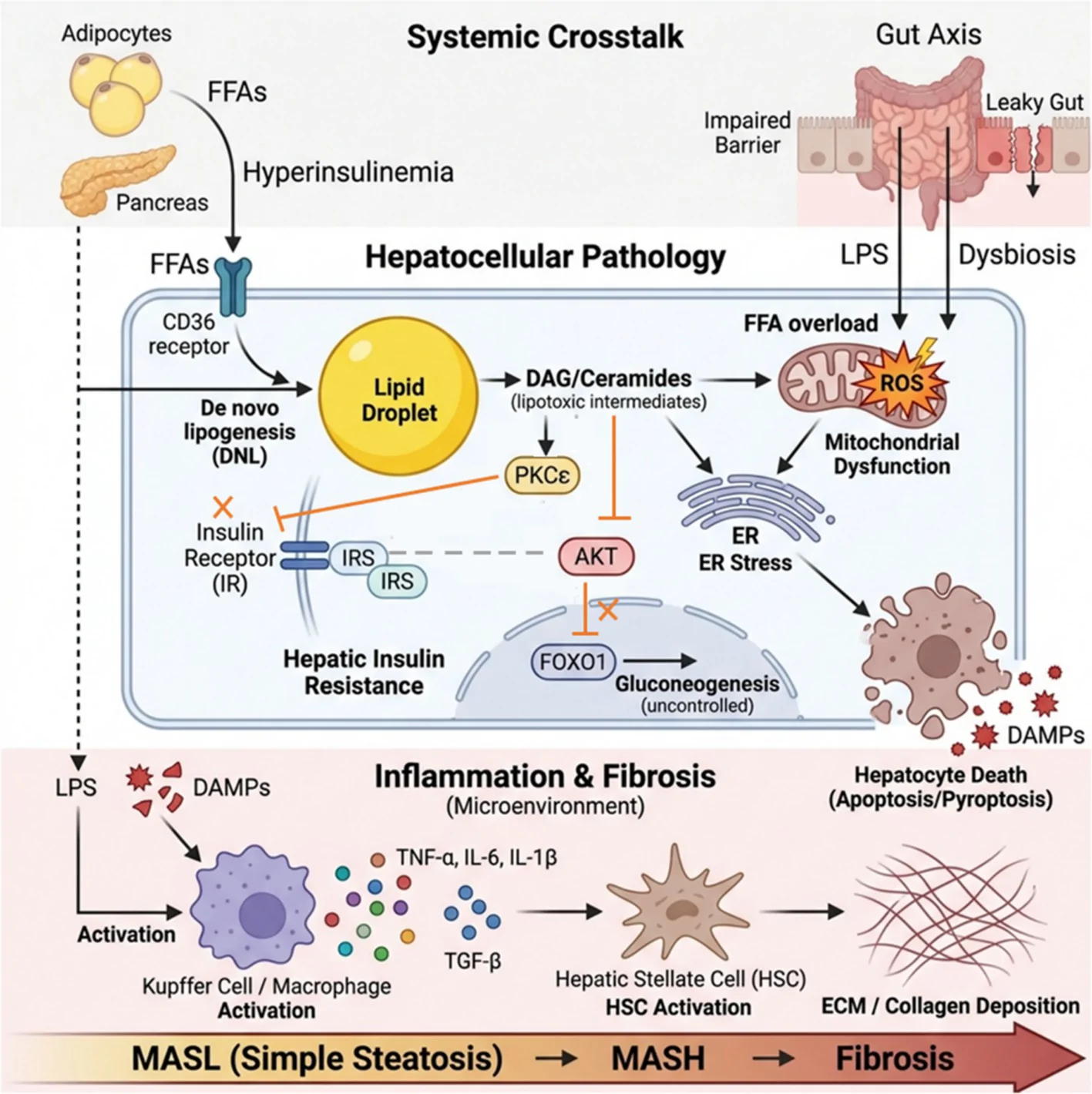
Pathogenesis of MASLD and progression to fibrosis.

Using bibliometric approaches, this review systematically synthesizes and comprehensively analyzes the pharmacological mechanisms, targeted therapeutic strategies, and clinical applications of berberine in MASLD. This analysis aims to furnish researchers with a comprehensive overview of the research landscape and emerging frontiers in this field, thereby providing an evidence based reference to guide future investigations.

#### 4.2.1 Molecular mechanisms and signaling pathways of berberine in regulating hepatic glucose and lipid metabolism

As revealed by our keyword-clustering and co-citation bibliometric outputs (Figure 7), molecular signaling governing hepatic glucose-lipid metabolism constitutes the largest and most well-established research cluster within the berberine-MASLD domain. AMPK/SIRT1 related signaling represents the most frequently reported canonical axis and forms the core of early research focus. Although multiple downstream effectors have been documented in experimental studies, our bibliometric analysis indicates that several newly identified molecular targets have not yet formed cohesive research sub-clusters. Against this quantitative background derived from our dataset, we systematically synthesize current mechanistic evidence regarding berberine-mediated modulation of hepatic glucose and lipid homeostasis.

Pathological mechanisms of glucose metabolic disorders in MASLD. Under physiological conditions, hepatic glucose homeostasis depends on the dynamic equilibrium and precise coordination of four metabolic components: endogenous hepatic glucose production, hepatic glycogen synthesis, glucose oxidation and utilization in hepatocytes, and the refined hormonal regulation of hepatic glucose output by insulin and glucagon (11). Disruption of this balance, manifesting as uncontrolled endogenous glucose production, blocked glycogen storage, restricted glucose oxidation, and failure of insulin to suppress hepatic glucose output, renders the liver trapped in a disorder characterized by glucose production exceeding consumption. Consequently, excess glucose and toxic metabolic intermediates abnormally accumulate within hepatocytes and are excessively released into the circulation. Surplus glucose and its derivatives trigger oxidative stress, endoplasmic reticulum (ER) stress, and inflammasome activation, leading to hepatocyte injury, apoptosis, and lobular inflammation. Persistent chronic inflammation further recruits macrophages, activates hepatic stellate cells, drives extracellular matrix deposition, and promotes the progression from simple steatosis to MASH, MASLD-F, and even HCC.

##### 4.2.1.1 Loss of inhibitory control over endogenous glucose production (EGP)

(1) Dual hyperactivity of gluconeogenesis and glycogenolysis. (i) In the gluconeogenic pathway, hyperactive lipolysis in peripheral and visceral adipose tissues releases large quantities of free fatty acids (FFAs) and glycerol into the liver via the portal vein (12). Intrahepatocellular β-oxidation of FFAs results in acetyl-coenzyme A (acetyl-CoA) accumulation, which allosterically activates pyruvate carboxylase (PC) and directly drives gluconeogenic carbon flux toward glucose synthesis (13). Meanwhile, as insulin signaling is impaired, the upstream transcription factor forkhead box protein O1 (FOXO1) is persistently localized in the nucleus, markedly inducing expression of the two rate-limiting enzymes phosphoenolpyruvate carboxykinase (PEPCK) and glucose-6-phosphatase (G6Pase), thereby promoting gluconeogenesis using lactate, glycerol, and alanine as substrates and resulting in substantial glucose synthesis (14, 15). (ii) At the glycogenolysis level, MASLD livers exhibit defective metabolic autoregulation: despite reduced hepatic glycogen reserves, glycogen phosphorylase maintains persistently high activity that is ineffectively suppressed by insulin (16). Under fasting conditions, concurrent hyperactivity of glycogenolysis and gluconeogenesis synergistically elevates basal hepatic glucose output (17, 18). Moreover, under physiological conditions, postprandial insulin phosphorylates FOXO1 via the phosphatidylinositol 3-kinase–protein kinase B (PI3K-AKT) pathway and promotes its nuclear export, thereby terminating gluconeogenic gene transcription (19–21). In MASLD livers, hepatic insulin resistance (Hep-IR) impedes AKT phosphorylation, resulting in persistent nuclear retention of FOXO1 and complete abolition of insulin-mediated EGP suppression (22). This impairment of postprandial hepatic glucose output suppression precipitates sustained fasting and postprandial hyperglycemia.

(2) Markedly impaired hepatic glucose uptake and glycogen synthesis capacity. The MASLD liver exhibits bidirectional impairment in the uptake and storage of circulating glucose. At the uptake stage, hepatocyte-specific glucokinase (GCK) activity is downregulated (23), compounded by the inhibitory effects of glucokinase regulatory protein (GCKR) gene polymorphisms (24), which attenuates the liver’s ability to sense postprandial hyperglycemia and prevents effective glucose entry into hepatocytes. At the storage stage, impaired insulin signaling leads to excessive phosphorylation and inactivation of glycogen synthase kinase 3 (GSK3), while glycogen phosphorylase remains persistently activated, impairing glycogen storage (25).

(3) Primary impairment of hepatocyte insulin signaling pathways: Hep-IR as the core upstream driver of glucose metabolic disorders in MASLD. The molecular mechanism underlying Hep-IR is characterized by post-receptor signaling damage mediated by lipotoxicity. Massive accumulation of lipotoxic intermediates such as diacylglycerol (DAG) and ceramide within hepatocytes activates protein kinase Cε (PKCε) (26, 27), which directly phosphorylates the kinase domain of the insulin receptor (IR) and inhibits its tyrosine kinase activity, thereby blocking downstream insulin receptor substrate (IRS)-PI3K-AKT signaling cascades (28–31). Studies have demonstrated that even mild elevations in intrahepatic triglyceride (IHTG) content can induce this post-receptor resistance, with the severity of resistance positively correlated with the degree of hepatic steatosis (32, 33). Furthermore, downregulation of carcinoembryonic antigen-related cell adhesion molecule 1 (CEACAM1) in MASLD hepatocytes reduces the efficiency of first-pass hepatic insulin clearance from the portal vein, triggering compensatory peripheral hyperinsulinemia (34). However, hyperinsulinemia not only fails to reverseHep-IR but also exacerbates the vicious cycle of “hyperinsulinemia–elevated hepatic glucose output–hepatic steatosis” by continuously stimulating hepatic lipogenesis.

(4) Pancreas–liver axis imbalance: pathological remodeling of endocrine feedback loops between pancreatic α-cells and the liver in MASLD. On one hand, On one hand, MASLD is accompanied by abnormal amino acid metabolism in skeletal muscle and the liver, leading to elevated levels of gluconeogenic amino acids such as alanine (35). These amino acids stimulate excessive glucagon secretion by activating the mechanistic target of rapamycin (mTOR) signaling pathway in α-cells, resulting in fasting hyperglucagonemia (36–38). On the other hand, hepatocellular glucagon receptor (GCGR) signaling becomes desensitized, manifesting as hepatic resistance to glucagon-mediated suppression of lipolysis and amino acid catabolism (39, 40). This sustains peripheral fat and amino acid catabolism, providing a continuous supply of precursors for hepatic gluconeogenesis. Simultaneously, persistent activation of the intracellular cAMP–protein kinase A (PKA) signaling pathway synergistically induces PEPCK and G6Pase expression via cAMP response element-binding protein (CREB) and FOXO1, further amplifying hepatic glucose production (41–44).

(5) Mitochondrial dysfunction and impaired oxidative metabolism. Mitochondrial oxidative metabolism abnormalities represent a critical organellar event linking early hepatic steatosis with advanced MASH-associated glucose metabolic disorders. In the early disease stage, the hepatic mitochondrial tricarboxylic acid (TCA) cycle undergoes compensatory upregulation (45); massive acetyl-CoA influx continuously drives gluconeogenesis via allosteric activation of PC (46). However, this explanation may be conceptually limited in human liver. Evidence suggests that aberrant upregulation of gluconeogenesis in MASLD is not driven by the total abundance of acetyl-CoA, but rather by dysregulated metabolic partitioning between its intracellular distribution and clearance pathways (47). As the disease progresses to MASH, mitochondrial respiratory chain complex function becomes compromised, and oxidative phosphorylation efficiency declines markedly (48). Consequently, hepatocytes are forced to rely on glycolysis for compensatory energy supply, leading to increased lactate generation (40). Large quantities of lactate return to the liver via the Cori cycle and are reused as gluconeogenic substrates, establishing a futile “glycolysis–gluconeogenesis” cycle that further elevates EGP. Meanwhile, impaired mitochondrial β-oxidation results in sustained FFA accumulation, aggravating DAG and ceramide mediated insulin resistance and creating a bidirectional vicious cycle between glucose and lipid metabolism at the mitochondrial level.

(6) Failure of gut–liver axis hormonal regulation: deficiency in glucagon-like peptide-1 (GLP-1) and fibroblast growth factor 19 (FGF19) signaling. In MASLD, distal gut hormones exhibit impaired negative regulation of hepatic glucose metabolism. Disruption of the secondary bile acid–intestinal farnesoid X receptor (FXR)/Takeda G protein-coupled receptor 5 (TGR5)–GLP-1 axis compromises glucose homeostasis and drives concurrent MASLD and Type 2 Diabetes Mellitus (T2DM) progression (49). Moreover, plasma GLP-1 levels inversely correlate with Homeostatic Model Assessment of Insulin Resistance (HOMA-IR), indicating attenuated enterohormonal control of glucose metabolism (50). High-fat diets and intestinal dysbiosis impair enteroendocrine L-cell function, leading to reduced postprandial GLP-1 secretion (51). GLP-1 suppresses hepatic EGP and promotes hepatic glycogen synthesis through indirect central–peripheral pathways. Its deficiency directly compromises postprandial glycemic control. Concurrently, the bile acid–FXR–FGF19 axis becomes disrupted. Altered bile acid profiles in MASLD patients result in insufficient intestinal FXR activation and markedly diminished FGF19 secretion (52). Under normal conditions, FGF19 acts on the liver via the systemic circulation to negatively regulate hepatic glucose production by suppressing FOXO1 and gluconeogenic rate-limiting enzyme expression (53, 54). Impairment of this pathway deprives the liver of critical distal negative feedback inhibition of EGP.

Pathological mechanisms of lipid metabolic disorders in MASLD. Under physiological conditions, intrahepatocellular triglyceride (TG) homeostasis relies on the dynamic equilibrium and precise coordination of four metabolic processes: exogenous fatty acid uptake, endogenous *de novo* lipogenesis (DNL), very-low-density lipoprotein (VLDL) assembly and secretion, and mitochondrial/peroxisomal fatty acid β-oxidation. Disruption of this balance constitutes a pathological trigger for MASLD development.

(1) Upregulated exogenous fatty acid uptake. In obesity and IR states, increased lipolysis in peripheral white adipose tissue (WAT) releases substantial quantities of non-esterified fatty acids (NEFAs) into the liver via the portal vein (12, 55, 56). Overexpression of fatty acid uptake transporters on the hepatocyte membrane, including members of the fatty acid transport protein (FATP) family (57) and scavenger receptor cluster of differentiation 36 (CD36) (58), significantly accelerates transmembrane transport of peripheral FFAs, providing abundant exogenous substrates for intrahepatic lipid deposition.

(2) Hyperactive hepatic DNL. Through sterol regulatory element-binding protein-1 (SREBP1) and carbohydrate-responsive element-binding protein (ChREBP) mediated signaling, insulin and glucose transcriptionally upregulate lipogenic enzymes, including acetyl-CoA carboxylase (ACC), fatty acid synthase (FAS), and stearoyl-CoA desaturase (SCD), thereby driving hepatic DNL. Additionally, the AMP-activated protein kinase (AMPK)-mTOR signaling network further modulates hepatic lipogenic capacity by regulating sterol regulatory element-binding protein-1c (SREBP-1c) activity (59, 60).

(3) Impaired VLDL assembly and secretion. Under physiological conditions, the liver assembles endogenous TG with apolipoprotein B-100 (ApoB-100) and phospholipids into VLDL particles, which are secreted into the circulation for delivery to peripheral tissues (61). In MASLD, this export pathway is compromised at multiple levels: reduced microsomal triglyceride transfer protein (MTTP) activity impairs ApoB-100 lipidation (62); The transmembrane 6 superfamily member 2 (TM6SF2) E167K variant impairs VLDL lipidation at the ER–Golgi interface, thereby reducing VLDL secretion (63, 64); and patatin-like phospholipase domain-containing protein 3 (PNPLA3) variants can also suppress VLDL secretion by interfering with lipid droplet remodeling (65). Collectively, these defects prevent effective efflux of intrahepatic TG, exacerbating hepatocellular steatosis.

(4) Impaired fatty acid β-oxidation. Under physiological conditions, the liver eliminates excess fatty acids through mitochondrial and peroxisomal β-oxidation pathways. In MASLD, attenuated peroxisome proliferator-activated receptor α (PPARα) expression and transcriptional activity suppress downstream target genes (e.g., carnitine palmitoyltransferase 1A (CPT1A)), thereby impairing hepatocellular fatty acid β-oxidation and exacerbating intrahepatic lipid accumulation (66–69).

(5) Increased cholesterol uptake. During MASLD pathogenesis, Niemann-Pick C1-like protein 1 (NPC1L1) regulates bidirectional cholesterol flux across the gut–liver axis (70, 71). Intestinal NPC1L1 promotes dietary cholesterol absorption, while canalicular NPC1L1 in the liver facilitates biliary cholesterol reuptake (72, 73). Together, these processes determine hepatic cholesterol load. Imbalance in this system leads to pathological cholesterol retention in hepatocytes, aggravating MASLD-associated steatosis and attendant metabolic stress.

(6) Activation of the *de novo* cholesterol synthesis pathway. Endogenous hepatic cholesterol biosynthesis is tightly controlled by rate-limiting enzymes, notably 3-hydroxy-3-methylglutaryl-coenzyme A reductase (HMGCR) and squalene epoxidase (SQLE). In the context of MASLD, upregulation of the sterol regulatory element-binding protein-2 (SREBP-2) transcriptional axis drives the overexpression of its downstream targets HMGCR and SQLE (74–76). Thereby accelerating the metabolic flux from acetyl-CoA to cholesterol and augmenting hepatic *de novo* cholesterol synthesis.

(7) Bile acid metabolic disorders. Bile acid synthesis, catalyzed by the rate-limiting enzyme cholesterol 7α-hydroxylase (CYP7A1) (77), represents the principal route for cholesterol catabolism and is aberrantly regulated in MASLD. Disruption of the FXR signaling pathway perturbs bile acid homeostasis (78). A key mechanism involves intestinal FXR-induced secretion of Fibroblast Growth Factor 15/19 (FGF15/19), which travels through the enterohepatic circulation to suppress hepatic CYP7A1 transcription. This gut–liver axis feedback is progressively impaired with MASLD-F (79), leading to reduced CYP7A1 expression and compromised cholesterol clearance. Ultimately, this dysregulation disrupts hepatic lipid and glucose metabolism (80), perpetuating disease progression.

Accumulating evidence has demonstrated that BBR interrupts the aforementioned pathological processes of lipid accumulation while concomitantly ameliorating excessive hepatic gluconeogenesis and systemic insulin resistance, thereby orchestrating multi-pathway synergistic correction of dual glucose–lipid metabolic disorders.

(1) Inhibition of excessive lipid uptake in hepatocytes. Under high-fat diet (HFD) stimulation, fatty acid transporters on the hepatocyte membrane, including CD36 and FATP5, are upregulated, mediating massive transmembrane influx of circulating FFAs and constituting the upstream initiating event of hepatic lipid deposition. Studies (81, 82) have shown that BBR significantly downregulates CD36 and FATP5 expression at both mRNA and protein levels, thereby reducing fatty acid influx. BBR primarily exerts remote regulation via the intestinal FXR–FGF15 gut–liver axis. By upregulating intestinal FXR activation, BBR promotes FGF15 secretion, which subsequently suppresses hepatic CD36 transcription through the portal circulation (83). Furthermore, at the organelle level, BBR can directly bind to Berardinelli-Seip congenital lipodystrophy 2 (BSCL2) protein, thereby inhibiting ER–lipid droplet membrane bridge formation and preventing aberrant expansion of nascent lipid droplets (84).

(2) Inhibition of hepatic DNL. SREBP-1c serves as the core transcription factor governing hepatic DNL, regulating downstream rate-limiting enzymes including ACC, FAS, and stearoyl-CoA desaturase 1 (SCD1). Existing literature indicates that BBR coordinately suppresses the SREBP-1c-centric lipogenic program through multiple upstream signaling pathways. (i) AMPK–SREBP-1c/2 classical pathway: Studies (85) have shown that 13-demethylberberine (DMB), the primary active metabolite of BBR, enhances hepatic AMPK phosphorylation in a dose- and time-dependent manner, suppresses the maturation and nuclear translocation of both SREBP-1c and SREBP-2, and reduces protein expression of their target genes, FAS and ACC, thereby blocking the conversion of acetyl-CoA to fatty acids and triglycerides at the source. Additional studies (86) have further confirmed that BBR activates AMPK and upregulates SIRT1, promoting phosphorylation-mediated inactivation of ACC at Ser79 and potently suppressing hepatic DNL. (ii) Clock/Bmal1 circadian rhythm pathway: Studies (87) have found that HFD suppresses expression of the hepatic core circadian heterodimer Clock/Bmal1, leading to uncontrolled transcription of downstream lipogenic genes. BBR significantly upregulates Clock/Bmal1, restoring circadian homeostasis and subsequently inhibiting SREBP-1c and lipogenic enzyme expression. RNA interference-mediated knockdown of Bmal1 completely abolishes the lipid-lowering effect of BBR, confirming that this pathway is indispensable for its therapeutic efficacy. (iii) SETD2 epigenetic modification pathway: Research (88) has revealed that BBR transcriptionally activates the histone methyltransferase SETD2, increasing the abundance of histone H3 lysine 36 trimethylation (H3K36me3), which epigenetically suppresses SREBP-1c promoter activity and downregulates lipogenic gene transcription. (iv) activating transcription factor 6 (ATF6)–SREBP-1c ER stress axis: Using a tunicamycin ™-induced acute ER stress model, studies (89) have demonstrated that BBR alleviates ER stress through chaperone activity, thereby inhibiting ATF6 nuclear translocation and its mediated activation of the SREBP-1c promoter, thus blocking ER stress-induced aberrant lipogenesis. (v) AKR1B10 as a direct protein target: Using cellular thermal shift assay (CETSA) and drug affinity responsive target stability (DARTS) techniques, studies (90, 91) have confirmed that BBR directly binds to and inhibits aldo-keto reductase 1B10 (AKR1B10). Inhibition of AKR1B10 subsequently upregulates PPARα and downregulates PPARγ, thereby suppressing SREBP-1c-mediated triglyceride and cholesterol synthesis.

(3) Promotion of fatty acid oxidation and lipid droplet catabolism in hepatocytes. CPT1A, located in hepatocyte mitochondria, is the rate-limiting enzyme for fatty acid β-oxidation. Under high-fat conditions, ubiquitination-mediated degradation of CPT1A is exacerbated, resulting in impaired fatty acid catabolism. Multiple pathways mediate BBR-induced stabilization of CPT1A and activation of lipolysis. (i) SIRT1–CPT1A deacetylation pathway: Studies (92) have found that under high-fat conditions, hepatocellular SIRT1 expression is downregulated, whereas acetylation levels at CPT1A Lys675 are elevated, accelerating its ubiquitin–proteasome-mediated degradation. BBR upregulates SIRT1 expression and enhances its deacetylase activity, specifically removing the acetyl modification at CPT1A Lys675, thereby inhibiting CPT1A ubiquitination and degradation, stabilizing and activating CPT1A protein, and promoting mitochondrial fatty acid oxidation (FAO). (ii) AMPK–peroxisome proliferator-activated receptor γ coactivator-1α (PGC-1α) oxidative pathway: Studies (85) have confirmed that DMB-mediated AMPK activation significantly upregulates PGC-1α, which coordinately upregulates the expression of mitochondrial CPT1A, acyl-CoA oxidase (ACO), and medium-chain acyl-CoA dehydrogenase (MC), thereby comprehensively enhancing the mitochondrial and peroxisomal oxidation rates of long-chain fatty acids. (iii) ERK–mTOR-dependent lipophagy: Studies (93, 94) have proposed an AMPK-independent lipophagy regulatory mechanism whereby BBR activates extracellular signal-regulated kinase 1/2 (ERK1/2), suppresses mechanistic target of rapamycin complex 1 (mTORC1) activity, induces autophagosome formation to encapsulate lipid droplets, and initiates lipophagy-mediated degradation. The autophagy inhibitor chloroquine (CQ) completely abrogates this lipid-lowering effect, demonstrating that lipophagy serves as an important complementary pathway for BBR-promoted lipid catabolism. (iv) PPARα-mediated lipid droplet remodeling: Studies (88) have shown that BBR-mediated PPARα activation downregulates lipid droplet fusion protein cell death-inducing DFFA-like effector a (CIDEA) and lipid droplet-associated proteins Perilipin 2 (PLIN2)/Perilipin 4 (PLIN4), inhibiting lipid droplet fusion and expansion. Concurrently, BBR directly binds to and inhibits BSCL2 protein, blocking ER–lipid droplet membrane bridge formation, thereby remodeling lipid droplet homeostasis at the organelle level and promoting intralipid triglyceride hydrolysis.

(4) Facilitation of hepatic lipid export. Hepatic lipid efflux primarily depends on two major pathways: reverse cholesterol transport (RCT) and very-low-density lipoprotein–triglyceride (VLDL–TG) secretion. BBR regulates key transporters involved in both processes. (i) MTTP–VLDL secretion pathway: Studies (85, 95) have confirmed that BBR upregulates MTTP expression at both protein and mRNA levels, restoring VLDL assembly and secretion and facilitating hepatic TG export. Concurrently, BBR upregulates low-density lipoprotein receptor (LDLR), accelerating hepatic uptake and degradation of circulating LDL-cholesterol (LDL-C), while downregulating apolipoprotein B (ApoB) expression to reduce abnormal lipoprotein generation, thereby synergistically ameliorating hyperlipidemia and hepatic lipid deposition. (ii) protein kinase Cδ (PKCδ)–ATP-binding cassette transporter A1 (ABCA1) reverse cholesterol transport pathway: Studies (96, 97) have found that BBR inhibits PKCδ activation, stabilizing hepatocyte membrane ABCA1 protein levels, promoting ABCA1–apolipoprotein A-I (apoA-I) interaction, accelerating cholesterol efflux, and reducing hepatic cholesterol accumulation.

(5) Amelioration of hepatic glucose metabolic disorders and insulin resistance by BBR. Excessive hepatic gluconeogenesis and insulin resistance represent core upstream drivers of MASLD. Using isotope tracer techniques, studies (98) have demonstrated that BBR significantly downregulates hepatic G6Pase and PEPCK expression, reduces hepatic glucose production rates, and improves HOMA-IR indices. Studies (87) have further confirmed that BBR restores Clock/Bmal1 circadian homeostasis, thereby inhibiting downstream FOXO1 nuclear translocation, suppressing gluconeogenic gene transcription, and reducing fasting blood glucose. Furthermore, studies (82) have demonstrated that BBR upregulates hepatocellular glucose transporter 2 (GLUT2) expression via the AMPK/SIRT1 axis, enhancing hepatic uptake and utilization of circulating glucose.

Although abundant pre-clinical studies have uncovered multiple signaling cascades through which berberine modulates hepatic glucose lipid homeostasis, these findings are predominantly derived from cell culture and rodent models. Critical uncertainties remain regarding whether these pre-clinical observations can be reproduced in human liver tissue, and how inter-individual metabolic heterogeneity may alter target responsiveness.

#### 4.2.2 Role and mechanisms of berberine in attenuating the pathological progression of MASLD from simple steatosis to MASH/hepatic fibrosis

Bibliometric clustering results (Figure 7) show that research focusing on MASLD progression, inflammation and fibrogenesis represents the second-largest research cluster in this field. Publications concerning oxidative stress, NLRP3 inflammasome and fibrotic signaling accumulate abundant citations. Nevertheless, bibliometric metrics suggest that most studies concentrate on post-onset therapeutic intervention, whereas investigations exploring early preventive effects against MASH progression remain relatively limited. Herein we summarize relevant mechanistic findings:

The first stage features insulin resistance driven hepatic lipid metabolic disorder, leading to simple hepatocellular steatosis. Upon this baseline, lipotoxicity, ER/mitochondrial stress, intestinal endotoxemia, innate immune activation, and genetic predisposition collectively establish a persistent second hit.

(1) Organelle stress and oxidative damage triggered by lipotoxicity.

(i) Mitochondrial dysfunction. Saturated free fatty acids (SFAs) directly impair the mitochondrial electron transport chain, resulting in massive reactive oxygen species (ROS) leakage, ATP depletion, and collapse of mitochondrial membrane potential (99). Concurrent suppression of the sirtuin 3 (SIRT3)–manganese superoxide dismutase (MnSOD) deacetylation pathway further compromises mitochondrial antioxidant defenses (100). If mitophagy is simultaneously impaired, ROS cannot be effectively eliminated, establishing a vicious cycle.

(ii) ER stress. Lipid overload triggers the unfolded protein response (UPR), concomitantly activating all three sensor pathways: the protein kinase R-like ER kinase (PERK)–eukaryotic initiation factor 2α (eIF2α)–activating transcription factor 4 (ATF4), inositol-requiring enzyme 1α (IRE1α)–X-box-binding protein 1 (XBP1), and ATF6 axes, which cooperatively upregulate the pro-apoptotic transcription factor C/EBP homologous protein (CHOP) (101). Sustained ER stress not only directly induces apoptosis but also aberrantly activates SREBP-1c through the ATF6 branch, thereby promoting DNL (102, 103).

(iii) Oxidative stress and ferroptosis. Insufficient activity of the nuclear factor erythroid 2-related factor 2 (Nrf2)/antioxidant response element (ARE) pathway, accompanied by decreased expression of heme oxygenase-1 (HO-1), NAD(P)H quinone dehydrogenase 1 (NQO1), and glutathione peroxidase 4 (GPX4), weakens the hepatocellular capacity to scavenge lipid peroxides (104–106). Under iron-overload conditions, iron-dependent accumulation of lipid ROS triggers ferroptosis, characterized by elevated prostaglandin-endoperoxide synthase 2 (PTGS2) and 4-hydroxynonenal (4-HNE) levels coupled with loss of plasma membrane integrity (107–109). This represents a novel form of regulated cell death that bridges simple steatosis and inflammatory injury.

(iv) Autophagy impairment. Defective hepatic autophagy results in impaired clearance of lipid droplets and damaged organelles, thereby exacerbating ER stress and IR (110, 111).

(2) Activation of innate and adaptive immune-inflammatory cascades. Following lipotoxicity-induced disruption of hepatocyte homeostasis, damage-associated molecular patterns (DAMPs) and pathogen-associated molecular patterns (PAMPs) cooperatively drive hepatic inflammation. (i) Toll-like receptor (TLR) and nuclear factor-kappa B (NF-κB) pathways. Disruption of the intestinal barrier permits portal delivery of Gram-negative bacteria-derived lipopolysaccharide (LPS) to the liver, activating Toll-like receptor 4 (TLR4)/myeloid differentiation primary response 88 (MyD88) on hepatocytes and Kupffer cells and subsequently triggering NF-κB and c-Jun N-terminal kinase (JNK) signaling, leading to massive release of tumor necrosis factor-α (TNF-α) and interleukin-6 (IL-6) (112, 113). A positive feedback loop between JNK1 and receptor-interacting protein kinase 3 (RIP3) continuously amplifies inflammatory signaling (114). (ii) Inflammasome and pyroptosis. Upon P2X7 receptor activation or mitochondrial ROS stimulation, the nucleotide-binding oligomerization domain-like receptor family pyrin domain-containing 3 (NLRP3)/apoptosis-associated speck-like protein containing a CARD (ASC)/Caspase-1 inflammasome assembles and cleaves Gasdermin D (GSDMD) to generate an N-terminal active fragment that forms pores in the plasma membrane (115–117). This process induces hepatocyte pyroptosis and releases mature interleukin-1β (IL-1β) and IL-18, thereby recruiting additional inflammatory cells (118). (iii) Macrophage recruitment and activation. Hepatocytes and damage-associated cells secrete chemokines such as C-C motif chemokine ligand 2 (CCL2), which recruit circulating monocyte-derived macrophages via C-C motif chemokine receptors including CCR2 and CCR5 (119, 120). These macrophages undergo metabolic reprogramming within the liver and further promote inflammation and fibrosis through secretion of IL-1β, TNF-α, and other pro-inflammatory mediators. (iv) Adaptive immunity and cellular signaling. Reduced hepatic Janus kinase/signal transducer and activator of transcription (JAK/STAT) protein tyrosine phosphatase activity enhances STAT1 signaling, driving T-cell infiltration and promoting fibrosis (121).

(3) During the MASH stage, persistent lipotoxicity, oxidative stress, and the inflammatory microenvironment activate hepatic stellate cells (HSCs), driving extracellular matrix (ECM) deposition. (i) HSC activation signals. The transforming growth factor-β1 (TGF-β1)/Smad canonical pathway represents the core driver of HSC activation, inducing expression of α-smooth muscle actin (α-SMA), collagen type I alpha 1 chain (COL1A1), and tissue inhibitor of metalloproteinases 1 (TIMP1). Simultaneously, high-mobility group box 1 (HMGB1) directly promotes HSC activation and fibrogenesis through the receptor for advanced glycation end-products (RAGE) (122). (ii) Macrophage–HSC crosstalk. Activated macrophages directly promote HSC activation through secretion of cytokines such as TGF-β (123) and platelet-derived growth factor (PDGF) (124), forming an “inflammation–fibrosis” feedforward loop.

In addition, the following aspects are also included. (4) Ductular reaction. Advanced MASH is accompanied by aberrant proliferation of biliary epithelial cells, which participate in ECM remodeling through secretion of pro-inflammatory and pro-fibrogenic signals (125). (5) Genetic susceptibility. Risk variants in genes such as PNPLA3 (126), TM6SF2 (127), membrane-bound O-acyltransferase domain-containing 7 (MBOAT7) (128), and hydroxysteroid 17-beta dehydrogenase 13 (HSD17B13) (129) can significantly accelerate progression from simple steatosis to MASH and fibrosis by influencing lipid droplet metabolism, inflammatory response thresholds, or fibrogenic susceptibility.

We have systematically organized and summarized studies regarding the effects and mechanisms of BBR in alleviating the progression of MASLD from simple steatosis to MASH and hepatic fibrosis from the following perspectives.

(1) Inhibition of lipotoxicity-induced oxidative stress and cell death:

(i) Nrf2/ARE endogenous antioxidant pathway. Studies (130) have demonstrated that BBR promotes the dissociation of Kelch-like ECH-associated protein 1 (Keap1) from Nrf2, driving Nrf2 nuclear translocation and its binding to the ARE, thereby upregulating the expression of HO-1, NQO1, and superoxide dismutase (SOD), and reducing hepatic ROS and malondialdehyde (MDA) levels. Further investigations (86) have revealed that BBR activates FOXO1 via SIRT1-mediated deacetylation, thereby upregulating antioxidant enzymes including SOD, glutathione peroxidase (GSH-Px), and catalase (CAT), and synergistically enhancing the hepatocellular antioxidant reserve.

(ii) Suppression of CYP2E1-mediated oxidative damage. Studies (10) have shown that the BBR active metabolite DMB and the derivative demethylenetetrahydroberberine (DMTHB) significantly downregulate the expression of the lipotoxic oxidase cytochrome P450 2E1 (CYP2E1), thereby reducing ROS generation at its source, elevating reduced glutathione (GSH) levels, and decreasing lipid peroxide MDA. (iii) Ferroptosis inhibition. Studies (95) have found that MASLD is accompanied by hepatic iron overload and activated ferroptosis. BBR upregulates cystine/glutamate transporter (SLC7A11) and GPX4, enhancing GSH reserves and suppressing the expression of ferroptosis markers such as 4-HNE and PTGS2, thereby concurrently ameliorating hepatic injury and secondary tissue lesions.

(2) Blockade of ER stress-mediated inflammatory initiation. The accumulation of free fatty acids and cholesterol triggers the UPR in hepatocytes; upon activation of the three branches, PERK, ATF6, and XBP1, pro-inflammatory gene transcription is driven. Studies (89) employing a tunicamycin-induced acute ER stress model have confirmed that BBR, through its chaperone activity, reduces the aggregation of denatured proteins and selectively inhibits the PERK–ATF4–CHOP pathway as well as ATF6 nuclear translocation, thereby alleviating ER dilation and injury. In multiple models including db/db and MCD diet models, BBR significantly reduces CHOP expression, blocking ER stress-driven release of TNF-α and IL-1β and its aberrant activation of SREBP-1c. Additional *in vitro* cellular studies (131) have further validated that BBR inhibits the PERK-ATF4-CHOP signaling axis, attenuating ER stress-induced hepatocellular damage.

(3) Multi-pathway blockade of hepatic chronic inflammation:

(i) JNK1 and TLR4/NF-κB inflammatory axis. High-fat diet-induced intestinal dysbiosis and metabolic endotoxemia allow LPS to enter the liver via the portal vein, activating TLR4 and its adaptor protein MyD88, which subsequently triggers NF-κB p65 nuclear translocation and massive release of TNF-α, IL-6, and IL-1β. Studies (132) have confirmed that BBR, independent of the AMPK pathway, significantly inhibits JNK1 phosphorylation in the liver and adipose tissue, thereby downregulating pro-inflammatory cytokine transcription. Concurrently, BBR reduces M1 pro-inflammatory macrophage infiltration into adipose tissue, blocking the delivery of adipose-derived inflammation to the liver via the circulation. Other studies (133, 134) have confirmed that BBR lowers plasma LPS levels by restoring the intestinal barrier and directly downregulates hepatocellular TLR4 and MyD88 expression, thereby suppressing NF-κB pathway activation and downstream inflammatory mediator release.

(ii) NLRP3 inflammasome pathway. Studies (10, 135) have shown that the BBR derivative DMTHB can directly bind to and target NLRP3, blocking inflammasome assembly of NLRP3/ASC/caspase-1, and reducing the cleavage and release of mature IL-1β and TNF-α. Additional studies (136) indicate that BBR blocks inflammasome activation upstream of NLRP3 by inhibiting purinergic receptor P2X7 activation; notably, the anti-inflammatory effect of BBR is abolished upon P2X7 gene knockout.

(iii) Chemokines and inflammatory cell recruitment. Studies (137) in ApoE^−^/^−^ mice have found that BBR restores the homeostasis between neutrophil elastase (NE) and its endogenous inhibitor alpha-1-antitrypsin (α1-AT), thereby suppressing chemokine CXCL12 and its receptor CXCR4 expression, blocking the downstream PI3K/Akt/NF-κB signaling cascade, and reducing neutrophil recruitment to the liver and hepatocyte ballooning degeneration. Another studies (138, 139) confirmed that BBR downregulates chemokine ligand 19 (CCL19) expression, blocking CCL19-TLR4-CCR2-mediated inflammatory cell infiltration. Yet another study (140) found that BBR downregulates the adipokine angiopoietin-like protein 2 (Angptl2), thereby inhibiting the Foxo1/NF-κB axis and balancing the regulatory T cell/T helper 17 cell (Treg/Th17) immune ratio, alleviating hepatic immune inflammation.

(4) Inhibition of HSC activation and fibrosis progression. HSC activation and collagen deposition represent the hallmarks of MASH progression to hepatic fibrosis. Studies (10, 135) have confirmed that the BBR derivative DMTHB significantly inhibits HSC activation and downregulates the expression of fibrosis marker proteins including TGF-β1, α-SMA, COL1A1, and TIMP1. Other studies (89) have also confirmed that BBR can inhibit HSC activation and collagen deposition. Furthermore, studies (95) in the STZ-HFHC combined model have revealed that BBR attenuates fibrosis by inhibiting the p38 mitogen-activated protein kinase (p38MAPK)/ERK-cyclooxygenase-2 (COX2) signaling axis; importantly, this intervention also blocks aberrant angiogenesis during the progression from fibrosis to hepatocellular carcinoma, thereby suppressing the malignant progression of MASLD throughout its entire course.

Cumulative pre-clinical data support berberine-mediated anti-oxidative, anti-inflammatory and anti-fibrotic activities; nevertheless, few studies translate these experimental organ level protective effects into clinically usable histological or serological endpoints for MASLD patients. The mismatch between rich pre-clinical mechanistic outputs and scarce clinical validation constitutes a notable translational bottleneck.

#### 4.2.3 Mechanistic studies of Berberine-mediated gut–liver axis regulation in MASLD

Citation burst analysis in CiteSpace (Figure 6) demonstrates that gut-liver axis associated topics represent one of the fastest-rising research clusters in recent years. Most existing publications focus on descriptive shifts of gut microbiota composition following berberine exposure, while causal mechanistic exploration linking specific microbial taxa to hepatic improvement is still limited. Within this bibliometric context, we synthesize current evidence for berberine mediated gut-liver axis regulation in MASLD:

Intestinal dysbiosis, disruption of the gut mucosal barrier, and aberrant microbial metabolites collectively constitute a persistent “exogenous hit” that drives MASLD progression. HFD increases the Firmicutes/Bacteroidetes ratio and promotes the overgrowth of Gram-negative bacteria (such as Enterobacteriaceae) that produce LPS (89), whereas the abundance of beneficial mucin-degrading bacteria, represented by *Akkermansia muciniphila*, and short-chain fatty acid (SCFA)-producing bacteria is significantly reduced (141, 142). Furthermore, intestinal dysbiosis directly compromises the physical barrier of the intestinal epithelium, providing persistent antigenic stimulation to the liver. HFD downregulates the expression of tight junction proteins, including zonula occludens-1 (ZO-1), occludin, and claudin-1, resulting in increased intestinal permeability (143). Luminal LPS (144), branched-chain amino acids (BCAAs) (145), endogenous ethanol (146), and viable bacteria translocate across the compromised intestinal barrier into the portal circulation, establishing chronic low-grade metabolic endotoxemia.

Translocated microbial components and metabolites subsequently disrupt hepatic homeostasis through multiple receptor–signaling axes.

(1) Microbial D-lactate as a pro-inflammatory metabolic messenger: Specific gut microbiota produce substantial quantities of D-lactate, which enters the liver through the portal circulation. Beyond serving as an energy substrate, D-lactate exerts immunomodulatory activity. It directly activates Kupffer cells and promotes neutrophil infiltration into the hepatic parenchyma via hepatic sinusoidal endothelial cell (Manifested as elevated myeloperoxidase (MPO) levels) activation (147), thereby significantly increasing the MASH activity score and hepatic inflammatory burden.

(2) Bile acid–nuclear receptor signaling disruption: Intestinal microbiota participate in bile acid metabolism via bile salt hydrolases (BSH), thereby regulating the primary-to-secondary bile acid ratio (148). Dysbiosis disrupts this metabolic network, suppressing the intestinal epithelial FXR–FGF15 signaling axis and consequently disturbing hepatic lipid synthesis, gluconeogenesis, and energy expenditure. Additionally, abnormal bile acid metabolism can affect nuclear receptor networks, including the liver X receptor (LXR), pregnane X receptor (PXR), and vitamin D receptor (VDR), synergistically exacerbating hepatocellular metabolic stress (149).

(3) SCFA deficiency and metabolic imbalance: SCFAs (e.g., butyrate) produced by beneficial bacteria through dietary fiber fermentation serve as a critical energy source for the colonic epithelium and function to enhance tight junctions (150), maintain barrier integrity, and modulate immunity. Under MASLD conditions, reduced SCFA production leads to insufficient nutritional support for the intestinal mucosa and impaired barrier repair capacity (151, 152). Concurrently, SCFA-mediated GLP-1 secretion is suppressed, further aggravating systemic insulin resistance (152).

The oral bioavailability of BBR is less than 1%, with the intestine serving as its primary site of enrichment. BBR remotely regulates hepatic glucose, lipid, and inflammatory homeostasis through three principal branches of the gut–liver axis: gut microbiota remodeling, intestinal barrier protection, and microbial metabolite signaling.

First, BBR reshapes the imbalanced gut microbiota architecture. MASLD models are characterized by an elevated Firmicutes/Bacteroidetes (F/B) ratio, enrichment of endotoxin-producing pathogenic bacteria, and reduced abundance of beneficial SCFA-producing bacteria. Studies (95) utilizing 16S rRNA sequencing have confirmed that BBR significantly increases the abundance of intestinal Bacteroidaceae and Bacteroides. Other studies (132) have demonstrated that BBR restores the high-fat-diet-induced reduction in the Bacteroidetes/Firmicutes ratio and recovers the abundance of beneficial bacteria such as Bifidobacterium and Lactobacillus. Further investigations (153) have confirmed that BBR upregulates the abundance of Firmicutes, Verrucomicrobia (*Akkermansia muciniphila*), and Lactobacillus; notably, the promotion of *A. muciniphila* does not occur through direct bacterial proliferation but rather indirectly by upregulating intestinal mucin protein mucin 2 (MUC2) and thickening the mucus layer, thereby improving its colonization environment. Studies (98) employing antibiotic-mediated microbiota depletion and fecal microbiota transplantation (FMT) experiments have established that the gut microbiota is an essential mediator of the therapeutic efficacy of BBR against MASH; the hepatoprotective effect of BBR is completely abolished upon microbiota clearance, whereas transplantation of feces from BBR-treated mice restores this therapeutic effect.

Second, BBR restores the intestinal mucosal barrier and ameliorates metabolic endotoxemia. High-fat diet disrupts ileal villous architecture and epithelial tight junctions, leading to increased intestinal permeability, translocation of Gram-negative bacteria-derived LPS into the bloodstream, and the establishment of metabolic endotoxemia. Studies (95) have shown that BBR significantly increases ileal villus height and crypt depth and reduces the bacterial translocation-positive rate. It has been demonstrated (133) that BBR upregulates the expression of the tight junction protein occludin, decreases the intestinal injury marker intestinal-type fatty acid binding protein (I-FABP), and reduces intestinal permeability. A study (154) in a largemouth bass model further validated that BBR upregulates tight junction proteins claudin-1, ZO-1, and occludin, reduces plasma diamine oxidase (DAO) levels, restores intestinal villous and microvillous architecture, and significantly inhibits intestinal cell apoptosis and ER stress.

In addition, BBR modulates microbial metabolite signaling pathways. (i) Intestinal FXR–FGF15 bile acid axis: Using wild-type and intestine-specific FXR-knockout (FXRint^−^/^−^) mice, studies (83) have confirmed that BBR inhibits gut microbial BSH activity, reduces primary bile acid deconjugation, and elevates intestinal concentrations of the endogenous FXR agonist TCA. TCA activation of the ileal FXR receptor induces secretion of FGF15. FGF15 reaches the liver via the portal vein, where it suppresses CD36-mediated fatty acid uptake, SREBP-1c-mediated lipogenesis, and NF-κB-mediated inflammatory signaling. (ii) SCFA and gut-derived hormone axis: Through microbiota functional prediction and metabolomic analyses, studies (132, 155) have found that BBR enriches butyrate- and propionate-producing gut bacteria (e.g., Roseburia and Blautia) and increases colonic SCFA content. SCFAs not only directly nourish the intestinal epithelium but also stimulate L cells to secrete GLP-1 and GLP-2, thereby improvingHep-IR and suppressing lipogenesis through endocrine pathways.

It remains unclear which specific bacterial taxa or microbial metabolites drive hepatic therapeutic benefits, as well as how host background modulates microbiota dependent treatment responses. The field is still dominated by descriptive associative observations rather than robust causal validations.

#### 4.2.4 Clinical research and formulation development of berberine for MASLD

Keyword evolution analysis from bibliometrix (Figure 8) reveals that formulation related keywords including nanoparticles solid dispersions and drug-delivery systems have become prominent rising hot topics whereas high quality clinical trial related publications remain comparatively scarce within this field. Below we summarize the status of clinical investigations and pre-clinical formulation development of berberine for MASLD:

A clinical evidence chain for BBR in the treatment of MASLD has been established, spanning monotherapy, combination therapy with synergistic enhancement, and large-sample validation across diverse populations, dosages, and treatment durations. A systematic search of PubMed and the WoSCC identified eight clinical trial-related articles (Table 5). We found that most studies administered BBR at 0.5 g three times daily (tid) for treatment courses of 12 to 18 weeks. Post-treatment outcomes included significant reductions in body weight and hepatic fat content, alongside improvements in liver function, serum lipids, blood glucose, and insulin resistance.

**Table 5:** Summary of clinical studies on berberine intervention in MASLD.

Study type	Tested ingredient	Study population	Sample	Intervention regimen	Primary outcome measures	Key efficacy results	Adverse events and safety	Main conclusions	References
Multicenter, randomized, double-blind, placebo-controlled Phase II clinical trial	HTD1801	Adults with suspected MASH/MASLD and T2DM.	100	BUDCA 500/1,000 mg BID or placebo, 18 wks; stable antihyperglycemic regimen maintained.	MRI-PDFF (liver fat), HbA1c, fasting insulin, ALT/AST, lipids, body weight.	High-dose group: significant reductions in liver fat, HbA1c, ALT.	Common adverse events: diarrhea, nausea; high-dose discontinuation rate ~12%.	High-dose BUDCA reduced hepatic fat, improved glycemic control, ALT in diabetic MASH; promising drug candidate	(156)
Randomized, double-blind, placebo-controlled clinical trial	BBR	Overweight/obese patients with MASLD (non-diabetic)	70	BBR 1,500 mg/day (500 mg tid) or placebo for 12 wks.	Liver enzymes, blood lipids, HOMA-IR, body composition, and hepatic fibrosis/steatosis indices (FIB-4/FLI).	Berberine reduced ALT, De Ritis ratio, and trunk fat.	Minority: mild, transient GI symptoms (flatulence, bitter taste)	BBR 1,500 mg/day: improved liver function, visceral fat, lipids, and inflammation in MASLD; no significant effect on fibrosis indices at 12 wks.	(157)
Multicenter, randomized, double-blind, placebo-controlled trial	Berberine	Obese patients with MASLD without diabetes	337	BBR 1 g/day or placebo for 6 months.	Relative visceral adipose tissue (VAT) change, absolute liver fat change (CT), lipids, glucose, hs-CRP.	VAT and liver fat unchanged; significant reductions in LDL-C, apolipoprotein B, and hs-CRP; higher baseline hs-CRP associated with greater lipid and anti-inflammatory benefits.	Comparable adverse event incidence across groups; minimal gastrointestinal reactions.	Obese MASLD without diabetes: 6-month berberine (1 g/day) safe but failed to reduce VAT and liver fat.	(158)
Single-center, randomized, double-blind, placebo-controlled clinical trial	Berberine	Overweight/obese patients with MASLD	70	BBR 1,500 mg/day (500 mg tid), or placebo for 12 wks.	Serum VEGF levels, arterial stiffness parameters: PWV (pulse wave velocity), PWA-SP (pulse wave analysis systolic pressure), AIx (augmentation index), fatty liver index (FLI), hs-CRP, etc.	BBR group: elevated VEGF, reduced PWA-SP; VEGF increase correlated with FLI improvement; age-adjusted PWV significant between groups, indicating better vascular elasticity	Mild, transient GI reactions (abdominal distension, bitter taste, flatulence)	12-week berberine (1,500 mg/day) upregulated VEGF and improved central BP/vascular function in MASLD; no effect on arterial stiffness.	(159)
Multicenter, randomized, double-blind, placebo-controlled Phase II clinical trial	HTD1801	Patients with suspected MASH comorbid with T2DM	100	HTD1801 500 mg or 1,000 mg BID, or placebo, for 18 wks.	Liver fat content by MRI-PDFF, cT1 fibro-inflammatory marker; HbA1c, liver enzymes, blood lipids, FIB-4 fibrosis index, etc.	High-dose group: 52% achieved MRI response, absolute liver fat reduction 4.8%; significant improvements in HbA1c, LDL-C, triglycerides, weight, ALT/GGT.	Most common: mild diarrhea, abdominal discomfort; few discontinued due to gastrointestinal reactions	HTD1801: improved liver histology markers, reduced hepatic fat, ameliorated glycemic/lipid metabolism and liver function in MASH.	(160)
Randomized, parallel-group, open-label clinical trial	Berberine	MASLD plus impaired glucose regulation (IGR) or T2DM	80	BBR 1.5 g/day + lifestyle intervention vs. lifestyle intervention (LSI) only, 16 wks.	Hepatic fat content (HFC) measured by ^1^H-MRS; blood glucose, blood lipids, liver enzymes; lipidomics.	BBR: reduced HFC, blood glucose, blood lipids, liver enzymes; lipidomics: decreased ceramide and C1P, modulated FFA, glycerophospholipid, and glyceride pathways.	No serious adverse events were reported.	Berberine reduced hepatic fat in MASLD, improved glycemic and lipid metabolism, and liver function; mechanism linked to serum lipid profile modulation, especially ceramide reduction.	(161)
Post-hoc analysis of a randomized, parallel-group, open-label clinical trial	Berberine, Pioglitazone	Adults with MASLD + IGR/T2DM	Total enrollment: 185; study completion: 155.	BBR 0.5 g tid + LSI vs. Pioglitazone 15 mg qd + LSI vs. LSI alone, 16 wks.	HFC measured by ^1^H-MRS; glycemic parameters, HOMA-IR, etc.	BBR + LSI: reduce hepatic fat; males showed greater improvement than females.	No serious adverse events were reported.	Greater benefits in male MASLD patients; sex-tailored treatment strategies warranted.	(162)
Multicenter, randomized, parallel-group, open-label clinical trial	Berberine, Pioglitazone	Adults with MASLD + IGR/T2DM	184	BBR 0.5 g tid + LSI vs. Pioglitazone 15 mg qd + LSI vs. LSI alone, 16 wks.	HFC measured by ^1^H-MRS; blood glucose, blood lipids, liver enzymes, etc.	Berberine reduced liver fat (efficacy comparable to pioglitazone); vs. lifestyle only: significant improvements in weight, HOMA-IR, lipids, and Apo-B; weight and lipid effects better than pioglitazone.	GI reactions (anorexia, gastric discomfort, diarrhea, constipation); 1 dropout (AE-related)	Berberine improved MASLD steatosis and metabolic parameters; superior to pioglitazone in weight reduction and lipid modification.	(163)

Although these studies have demonstrated the therapeutic potential of BBR for MASLD, its clinical application remains constrained by several significant limitations. (1) Poor oral absorption and extremely low bioavailability: As a quaternary ammonium alkaloid, BBR exhibits poor lipid solubility, and intestinal efflux transporters limit its oral bioavailability to less than 1%. Yan et al. reported a post-treatment serum concentration of 6.99 ng/mL (163); although the hepatic concentration is approximately 50–70 fold higher than plasma levels (157), it remains far below effective concentrations required *in vitro*. (2) Safety and compliance issues: The most common adverse reactions include appetite suppression, abdominal discomfort, and diarrhea, which predominantly occur during the initial treatment phase. Although generally tolerable, these effects compromise long-term adherence. (3) Lack of standardized dosing and duration: Effective doses range from 1,000 to 1,500 mg/day; however, higher doses are associated with increased gastrointestinal adverse reactions. Most studies have been limited to ≤24 weeks, and long-term (≥1 year) efficacy and safety data are lacking. (4) Population heterogeneity in therapeutic response: A recent RCT enrolling 337 non-diabetic obese MASLD patients demonstrated that BBR (1 g/day for 6 months) failed to significantly reduce visceral fat area or hepatic fat content, suggesting that BBR efficacy may depend on the degree of baseline metabolic disturbance (158).

Although these limitations indicate that the clinical translation of BBR requires further optimization, some questions may be clarified through larger-scale RCTs with longer follow-up periods, including optimal dose ranges, long-term efficacy, and response differences across metabolic backgrounds. However, to overcome the physicochemical limitations of poor water solubility, weak intestinal transmembrane permeability, and extremely low oral bioavailability, diverse nano-delivery systems and novel dosage forms have been engineered. Notably, all of these formulations are currently confined to in-vitro and pre-clinical animal studies; none have progressed to human clinical trials. These strategies focus on four principal directions: enhancing solubility, improving gastrointestinal stability, promoting intestinal mucosal permeation, and achieving hepatic targeting, thereby maximizing it’s *in vivo* pharmacological effects.

(1) Diethylaminoethyl-dextran (DEAE-DEX)–modified bile salt hybrid liposomes. DEAE-DEX as an outer shell to co-encapsulate BBR and curcumin, this system resists acid–base degradation in the digestive tract (164). Bile salts reversibly open intestinal tight junctions to facilitate transmembrane absorption, while hepatocyte asialoglycoprotein receptor (ASGPR)–mediated targeting enables hepatic enrichment. At equivalent doses, the formulation exhibits 34-fold greater pharmacological efficacy than free BBR, activating the Nrf2 antioxidant pathway and inhibiting the NLRP3 inflammasome, thereby significantly ameliorating hepatic steatosis in HFD-induced mice.

(2) Bovine serum albumin nanoparticles (BBR-BSA NPs). Studies (93, 165) have employed BSA-loaded BBR nanoparticles. At only one-tenth the equivalent dose of free BBR, the nanoparticles significantly improved hepatic lipid accumulation, necrotic inflammation, and early-stage fibrosis. Mechanistically, the nanoparticles enhance gastrointestinal stability and hepatic targeting enrichment, activate sigma-1 receptor (SIGMAR1)–mediated ER homeostasis and the mTOR-dependent lipophagy pathway, and inhibit SREBP-2 mediated cholesterol synthesis. The lipid-lowering and anti-fibrotic effects surpass those of free BBR and the positive control atorvastatin.

(3) Gliadin–chitosan nanoparticles. Studies (166) have utilized food-grade gliadin hydrolysate–chitosan to encapsulate BBR, significantly improving its water solubility and mucus-penetrating capacity. Hepatic transcriptomic and metabolomic analyses in HFD-fed mice confirmed that these nanoparticles coordinately modulate fatty acid oxidation, lipogenesis, cholesterol metabolism, and bile acid metabolic pathways. At equivalent doses, the magnitude of reduction in hepatic lipid deposition and oxidative stress was significantly superior to that of free BBR, indicating substantial potential for industrial development.

(4) Solid lipid nanoparticles (SLNs). SLNs substantially enhance the oral bioavailability of BBR and facilitate passive hepatic targeting, thereby augmenting its therapeutic potential for liver-associated diseases. Fabricated via well-established methodologies, such as ultrasonication combined with spray drying, SLNs yield solid powders characterized by uniform particle size, high encapsulation efficiency, and favorable redispersibility and stability, indicating strong potential for industrial scale-up (167). Moreover, the sustained-release properties of SLNs offer a promising strategy to circumvent the gastrointestinal adverse effects associated with high-dose administration of free BBR, which is necessitated by its inherently poor oral bioavailability (168).

(5) Liposomes and long-circulating liposome systems. Although conventional phospholipid liposomes improve BBR solubility and enable passive targeting to reticuloendothelial system (RES)-rich organs such as the liver, they are hampered by short systemic circulation half-lives and pronounced burst release. To address these limitations, surface PEGylation has been employed to construct long-circulating liposomes. The hydrophilic PEG layer creates a steric barrier that reduces opsonization and RES-mediated clearance, thereby significantly extending their plasma residence time (169).

(6) Microsphere formulations. Berberine microspheres fall into natural polymer, synthetic polymer and composite types. Carriers like chitosan, gelatin, alginate and beta-cyclodextrin (β-CD) feature good biocompatibility, degradability and auxiliary antioxidant/antibacterial activity, but fabrication methods cause big differences in drug loading and particle uniformity. Emulsion-crosslinked chitosan microspheres have high drug loading, while membrane emulsification yields microspheres with stable size and release.

(7) Solid dispersion technology. Solid dispersion is a key strategy to raise berberine oral bioavailability. It disperses BBR as amorphous/molecular form in polymers to boost dissolution and stability. Water-soluble polyvinylpyrrolidone (PVP) (170) combined with surfactants accelerates drug dissolution. Solvent-free hot-melt (171, 172) extrusion with enteric carriers achieves intestinal targeted delivery and higher bioavailability. Composite systems of phospholipid, TPGS and silica (173) work together to improve intestinal permeability and block P-gp efflux. Rational matching of carriers and processes synergistically solves BBR’s poor oral absorption from solubilization, permeation and efflux inhibition perspectives.

Despite encouraging pre-clinical pharmacological outcomes, multiple critical obstacles impede the clinical translation of berberine nano-formulations. First, most preparation protocols are optimized for laboratory small batch synthesis. Even for formulations considered promising for industrial development, standardized large scale manufacturing processes are largely absent, and batch to batch uniformity in particle size, drug loading efficiency and release behavior remains difficult to guarantee. Second, existing in-vivo evaluations are mostly short term animal experiments; data regarding chronic organ accumulation, repeated dose long term safety, and off target toxicity are scarce. Third, many systems exhibit only passive hepatic enrichment rather than genuine active receptor mediated targeting, resulting in insufficient targeting specificity toward diseased hepatocytes or activated hepatic stellate cells. Fourth, certain carrier materials, special modification procedures and purification steps raise overall production costs, restricting practical popularization. Finally, unified quality control specifications for berberine nano-preparations have not yet been established, including accepted testing criteria for particle size distribution, encapsulation efficiency, stability and impurity limits. Collectively, these bottlenecks indicate that substantial challenges remain before these promising pre-clinical nano-formulations can be translated for human use.

### 4.3 Strengths and limitations

Despite employing scientifically rigorous and well established methodologies to investigate the current landscape of berberine for MASLD, this study possesses several strengths: Database selection and analysis: We separately analyzed bibliometric data retrieved from WoSCC and Scopus, as well as clinical trial related records from PubMed. This strategy achieved relatively sufficient data coverage while avoiding the risk of impaired citation analysis performance caused by direct merging of datasets from different databases. Nevertheless, several important limitations of this work should be acknowledged: (1) Language related bias and publication bias. Only English language publications were included in this study. This setting may introduce both language bias and publication bias: high quality studies published in other languages were excluded. (2) Limitations of database coverage. Although WoSCC, Scopus and PubMed are widely used academic databases, they cannot capture all relevant evidence. (3) Timeliness limitation from the fixed time cutoff. Bibliometric analysis relies on a predefined retrieval time cutoff. Newly published papers after this cutoff date were not incorporated, and recent articles have not yet accumulated sufficient citation records; accordingly, the latest emerging research hotspots may be under represented. (4) Author level analysis was not performed. We did not conduct quantitative analysis regarding authors, research teams or institutional collaborations, mainly because name homonymy and highly similar author names frequently occur in bibliometric datasets, which would substantially increase the risk of mis-attribution and analytical error. (5) Inherent limitations of bibliometric indicators. Citation frequency and other bibliometric metrics were used to evaluate research influence. However, citation counts cannot completely represent the intrinsic scientific quality or clinical significance of a single publication. Despite these objective limitations, the overall conclusions of this bibliometric analysis remain reliable and can provide useful reference for future basic and clinical research focusing on berberine against MASLD.

### 4.4 Research frontiers and translational challenges

Building upon the preceding comprehensive summary and analysis of berberine’s pharmacological mechanisms and clinical applications in MASLD grounded in our bibliometric outputs, this section explicitly describes the unique knowledge gaps, mechanistic insights, and translational implications yielded by the present synthesis. Moving beyond descriptive recapitulation of published literature, we highlight several emerging research frontiers that have been objectively recognized from bibliometric mining, and further put forward an evidence-based tiered research agenda with concrete actionable priorities for future investigations.

(1) The gut-liver axis represents a rapidly expanding research frontier. According to our bibliometric findings, research attention toward gut microbiota-related topics has increased sharply since 2020. Most existing studies merely describe compositional shifts in gut microbiota following berberine intervention, whereas causal evidence linking specific bacterial taxa or metabolites to hepatic pathological improvement remains limited. Priority agenda: (i) deploy multi-omics integration in longitudinal MASLD cohorts to delineate temporal causal chains linking microbiota, metabolites, and histology; (ii) validate candidate taxa via fecal microbiota transplantation and germ-free mouse models; and (iii) develop predictive microbial signatures of berberine response to enable microbiome-guided therapeutics.

(2) Stratified precision clinical research represents a developing frontier. Thematic evolution maps indicate a gradual shift toward clinical validation in this field since 2024. Current clinical evidence derives predominantly from small-scale, short-duration trials. Although published randomized controlled trials have observed interindividual heterogeneity in therapeutic response, stratified studies incorporating baseline metabolic phenotypes, MASLD disease stages, and genetic backgrounds remain severely underrepresented. Priority agenda: (i) design adequately powered randomized controlled trials with prespecified stratification by metabolic phenotype, fibrosis stage, and major risk genotypes; (ii) employ validated non-invasive biomarkers as pharmacodynamic endpoints to enhance statistical power; and (iii) establish real world evidence platforms to characterize long-term efficacy, safety, and withdrawal effects across diverse ethnic populations.

(3) Formulation innovation addressing bioavailability bottlenecks constitutes another promising frontier. In our analysis, nanoparticles, solid dispersions, and drug delivery systems have emerged as rising hot keywords. Multiple berberine formulations have demonstrated favorable efficacy in preclinical animal models, yet most remain at the proof-of-concept stage. Priority agenda: (i) conduct standardized Phase I/IIa pharmacokinetic studies in healthy volunteers and MASLD patients; (ii) evaluate long-term toxicological profiles of nano-carriers; and (iii) prioritize the development of liver-targeted platforms, such as bile acid-modified nanoparticles, using MASLD-specific histological regression as an efficacy benchmark for comparison against standard berberine.

(4) Multi-target combination therapy represents an emerging exploratory frontier. Leveraging berberine’s polypharmacological properties, preclinical studies have increasingly explored its combination with metformin, statins, GLP-1 receptor agonists, and structured lifestyle interventions. Despite promising synergistic signals in animal models, rigorous clinical evidence is currently absent. Priority agenda: (i) utilize network pharmacology and human liver organoid models to deconvolute synergistic mechanistic networks and screen optimal drug combinations; (ii) conduct factorial design randomized controlled trials comparing berberine combination therapy, monotherapy, and standard of care alone; and (iii) formulate disease stage specific strategies, for example, comparing berberine as early phase insulin sensitizing priming therapy versus late-phase anti-fibrotic maintenance therapy.

Although the abovementioned frontiers are highly promising, multiple practical bottlenecks continue to impede the translation of basic research into clinical application. At the clinical level, major obstacles include a lack of large-scale, long-term randomized controlled evidence; undefined optimal dosing regimens; incompletely elucidated sources of interindividual heterogeneity in therapeutic response; and insufficient long-term safety data. At the mechanistic level, the vast majority of inferences are derived from cellular and rodent experiments, with a paucity of human-validated molecular targets and causal evidence for gut-liver axis effects, rendering preclinical findings difficult to directly extrapolate to human MASLD pathophysiology. At the formulation level, although various berberine nano-delivery systems have demonstrated favorable preclinical efficacy, multiple practical obstacles regarding manufacturing scalability, safety profiles, targeting specificity, and quality control hinder their clinical advancement (see Section 4.2.4 for details).

Collectively, these frontiers delineate an evidence-based translational roadmap for berberine in MASLD. The field must transition from associative microbiota surveys to causal mechanistic investigations, from small, homogeneous trials to phenotype-stratified precision randomized controlled trials, from rodent-limited formulation concepts to clinical pharmacology programs, and from descriptive monotherapy reports to combination studies.

## 5 Conclusion

This review integrates bibliometric quantitative analysis with systematic literature synthesis to objectively delineate the research landscape of berberine in MASLD, identifying core research hotspots and data-driven directions for future investigation. Accumulated evidence demonstrates that berberine exerts multi-target, multi-pathway synergistic effects via AMPK/SIRT1, FXR-FGF15, Nrf2/ARE, NLRP3, and JNK/NF-κB, thereby correcting hepatic glucolipid metabolic disorders, suppressing oxidative stress and chronic inflammation, and arresting fibrotic progression. Additionally, berberine achieves remote hepatic regulation through the gut microbiota-metabolite-liver axis, significantly ameliorating or even reversing the complex pathological manifestations of MASLD. Nevertheless, substantial practical limitations persist, including extremely low oral bioavailability, pronounced inter-individual heterogeneity in therapeutic response, and a lack of long term safety data, all of which continue to hinder clinical translation. Therefore, future research should prioritize elucidating the causal roles of the gut-liver axis and identifying predictive microbial biomarkers of treatment response; conducting stratified, high-quality clinical trials to strengthen real world evidence; advancing druggability and safety evaluations of targeted berberine delivery systems; and exploring rational combination therapeutic strategies based on disease staging. Collectively, these efforts are essential to facilitate the transition of berberine from a basic research candidate to a precise, individualized therapeutic agent for MASLD.

## Data Availability

The original contributions presented in the study are included in the article/Supplementary material. Data will be made available on request. Data from the Web of Science database, PubMed database. Available through the following URL: http://www.webofscience.com, http://www.scopus.com, and https://pubmed.ncbi.nlm.nih.gov/.
