# Supplementary material for: Berberine and metabolic dysfunction-associated steatotic liver disease: a global analysis of molecular mechanisms, translational challenges and research frontiers
Source: Front Nutr. 2026 Oct 8;13:1969017. doi: 10.3389/fnut.2026.1969017 (PMC13687011; doi:10.3389/fnut.2026.1969017)
Supplement: Supplementary file 1 [file Table_1.docx]

**Web of Science database search formula**

(((TS=(Berberine*)) AND TS=("metabolic dysfunction-associated steatotic liver disease" OR MASLD OR "metabolic dysfunction-associated fatty liver disease" OR MAFLD OR "nonalcoholic fatty liver disease" OR NAFLD OR "metabolic dysfunction-associated steatohepatitis" OR MASH OR "nonalcoholic steatohepatitis" OR NASH OR "nonalcoholic fatty liver" OR NAFL)) AND LA=(English)) AND DOP=(2012-01-01/2026-04-01)) AND DT=(Article OR Review) (Note: TS=Topics; LA=Language; DOP=Publication date; DT=Document type).

**PubMed database search formula**

(("Berberine*"[Title/Abstract] AND ("metabolic dysfunction-associated steatotic liver disease"[Title/Abstract] OR "MASLD"[Title/Abstract] OR "metabolic dysfunction-associated fatty liver disease"[Title/Abstract] OR "MAFLD"[Title/Abstract] OR "metabolic dysfunction-associated steatohepatitis"[Title/Abstract] OR "MASH"[Title/Abstract] OR "nonalcoholic steatohepatitis"[Title/Abstract]) OR "NASH" OR "nonalcoholic fatty liver" OR "NAFL")) AND (("2012/01/01"[Date - Publication] : "2026/04/01"[Date - Publication])) Filters: Clinical Trial, English

**Scopus database search formula**

TITLE-ABS-KEY (Berberine*) AND TITLE-ABS-KEY ("metabolic dysfunction-associated steatotic liver disease" OR MASLD OR "metabolic dysfunction-associated fatty liver disease" OR MAFLD OR "nonalcoholic fatty liver disease" OR NAFLD OR "metabolic dysfunction-associated steatohepatitis" OR MASH OR "nonalcoholic steatohepatitis" OR NASH OR "nonalcoholic fatty liver" OR NAFL) AND PUBYEAR > 2008 AND PUBYEAR < 2027 AND (LIMIT-TO (DOCTYPE , "re") OR LIMIT-TO (DOCTYPE , "ar")) AND (LIMIT-TO (LANGUAGE , "English"))
